# Enhancing the Reaction of CO_2_ and H_2_O Using Catalysts within a Nonthermal Plasma

**DOI:** 10.1021/acscatal.5c00747

**Published:** 2025-04-16

**Authors:** Piu Chawdhury, Sarayute Chansai, Matthew Conway, Joseph Parker, Matthew Lindley, Cristina E. Stere, Meenakshisundaram Sankar, Sarah J. Haigh, Ben Dennis-Smither, Sorin V. Filip, Stephen Poulston, Peter Hinde, Christopher Hawkins, Christopher Hardacre

**Affiliations:** 1 Department of Chemical Engineering, The University of Manchester, Oxford Road, Manchester M13 9PL, U.K.; 2 Cardiff Catalysis Institute, School of Chemistry, Cardiff University, Maindy Road, Cardiff CF24 4HQ, United Kingdom; 3 Department of Materials, The University of Manchester, Manchester M13 9PL, United Kingdom; 4 Low Carbon Innovation Centre, BP International Ltd, Saltend Chemicals Park, Hull HU12 8DS, U.K.; 5 BP Technology Centre, Whitchurch Hill, Pangbourne RG8 7QR, U.K.; 6 Johnson Matthey Technology Centre, Blount’s Court, Sonning Common, Reading RG4 9NH, U.K.; 7 JM Technology Centre, Chilton Site, Belasis Avenue, Billingham TS23 1LB, U.K.

**Keywords:** nonthermal plasma (NTP) catalysis, CO_2_ and H_2_O conversion, H_2_ production, Cu catalysts, metal−support interaction, *in situ* DRIFT-MS

## Abstract

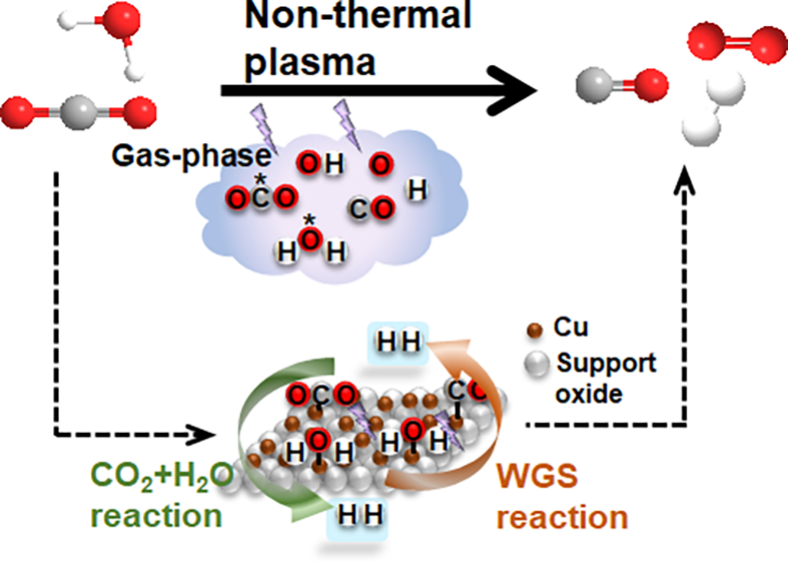

The direct conversion of emitted and captured carbon dioxide into usable fuels remains a significant challenge and is a key element in the transition to net zero. Herein, we examine the reaction of CO_2_ and H_2_O over Ni- and Cu-based catalysts combined with nonthermal plasma (NTP) technology. The catalysis under NTP conditions enabled significantly higher CO_2_ conversion and product yield, which was almost six times higher than that of the plasma-only system. A maximum H_2_ concentration of ∼2500 ppm was achieved for the Cu/ZSM5 catalyst at 17% CO_2_ conversion. Comprehensive catalyst characterization together with the reaction performances reveals that Cu in a reduced state promotes both the CO_2_ and H_2_O conversion leading to H_2_ formation. *In situ* diffuse reflectance infrared spectroscopy (DRIFTS) coupled with mass spectrometry (MS) analysis of the gas phase products confirms that CO is the major active species to drive the water gas shift reaction to form H_2_ in addition to the direct CO_2_ and H_2_O interaction. It also explains how the different metal support interactions influence the CO adsorption and its interaction with water. Among the catalysts studied, ZSM5-supported Cu catalysts were found to be the most effective in facilitating the CO_2_ and H_2_O reaction to produce H_2_.

## 1 Introduction

Mitigation of greenhouse gas emissions has become of unprecedented importance due to the growing global climate crisis caused by the rapid increase of CO_2_ and CH_4_ in the atmosphere.^1,2^ However, CO_2_ is also a ubiquitous and commonly available C1 feedstock resource around the world. Hence, a promising strategy to reduce this global problem is the conversion and utilization of captured carbon dioxide, which can reduce the CO_2_ emissions and, at the same time, produce valuable fuels and chemicals for energy storage.^3−5^ However, CO_2_ is a very stable molecule and often requires high temperatures and/or pressures coupled with catalytic systems to enable its conversion to value-added products. Although the conversion or selective reduction of CO_2_ to useful fuels using noncarbon-based energy sources (such as solar, wind, nuclear, or geothermal) is expected to be a sustainable alternative to reduce CO_2_ emission, the challenge is to make the overall process energy efficient and cost-effective. Commonly, H_2_ is used as coreactant for the conventional CO_2_ hydrogenation to fuel synthesis, but in nature, water is used as the hydrogen source through the process of photosynthesis. Furthermore, water is commonly emitted with CO_2_ in industrial processes such as ammonia production and, hence, technologies that aim to convert CO_2_ immediately at the exit of industrial installations could use the water as a coreactant of CO_2_. Therefore, the direct conversion of CO_2_ and H_2_O to value-added products would be a promising approach based on the use of cheap, abundantly available raw materials.

The reaction of CO_2_ and H_2_O is thermodynamically unfavorable due to the chemical stability of both components (eq 1).
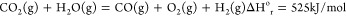
1

Electrocatalysis and photocatalysis have both been studied for this process but have limitations due to the operational temperature, in the case of electrocatalysis,^6^ and the low efficiency of solar energy utilization in photocatalysis.^7^ Recently, nonthermal plasma (NTP) technology has been gaining attention in this field, enabling CO_2_ activation at low temperature and atmospheric pressure. Nonthermal plasmas can be initiated under ambient conditions and operated at temperatures ranging from room temperature to several hundred Kelvin. The average electron temperature in cold plasmas is typically 1–10 eV, which results in activating the reactant molecules through vibrational and electronic excitation, while keeping the gas phase kinetic temperature low.^8−10^ Moreover, considering the fact that nonthermal plasma is powered by electricity, which can be generated from renewable sources such as solar and wind, NTPs would enable CO_2_ molecules to be incorporated into a renewable carbon cycle that can reduce our dependence on fossil fuels. In addition, this method is suitable for decentralized and relatively small-scale CO_2_ conversion, providing a means to couple the process with a range of CO_2_ sources directly at suitable locations without the need for storage and transportation, for example. A significant amount of research has been reported on the plasma-activated CO_2_ conversion via CO_2_ hydrogenation^11−15^ or dry reforming reactions;^16−20^ however, to date, few studies have reported simultaneous conversion of CO_2_ and H_2_O into syngas or oxygenates either in the absence (plasma only) or presence of a catalyst.

Ihara et al. were the first to investigate the conversion of CO_2_ and H_2_O using a microwave plasma-only reaction, wherein it was found that oxalic acid and H_2_O_2_ were formed as the main liquid products following condensation in a cold trap after 1 h of plasma reaction.^21^ Snoeckx et al. studied the utilization of a dielectric barrier discharge (DBD) plasma-only condition for the CO_2_–H_2_O reaction into value-added products.^22,23^ Therein, the main products observed were H_2_, CO, O_2_, and H_2_O_2_. Chen et al. employed surface-wave and low-pressure microwave plasma-only systems for the CO_2_–H_2_O conversion resulting in syngas as the major product.^24,25^ Hayashi et al. investigated the CO_2_–H_2_/H_2_O conversion using a surface discharge forming CO, CH_4_, and dimethyl ether as products. They also observed that the addition of water to the feed lowered the CH_4_ formation rate compared to when H_2_ was added.^26^ The reaction of CO_2_–H_2_O to ethanol under a negative DC corona discharge was studied by Guo et al. and highlighted that the electron attachment process that created accelerated anions of CO_2_ and H_2_O was important.^27^ In addition to the plasma-only systems, NTPs coupled with catalysts have also recently been recently reported. Ma et al. observed that CO_2_ and H_2_O conversion significantly increased using a Ni/γ-Al_2_O_3_ catalyst coupled with a DBD plasma resulting in an improved syngas ratio and methane formation compared with the plasma-only system.^28^ Yao et al. also showed similar plasma-catalytic results over a NiO-based catalyst coupled with a DBD plasma system resulting in a maximum H_2_ concentration of 1022 ppm being formed.^29^ The conversion of the CO_2_/H_2_O mixture over a TiO_2_-supported NiO catalyst in a pulsed surface-wave sustained microwave discharge was investigated by Chen et al. They highlighted that oxygen vacancy formation on the catalytic support was a major factor for improved CO_2_ conversion.^25^ However, understanding the mechanism and structure–activity/selectivity relationships for NTP-CO_2_/H_2_O conversions is still limited. Therefore, more fundamental work is still required to optimize the reaction condition, finding suitable catalyst combination that can optimize the overall process energy efficiency while uncovering the underlying mechanism that drives the reaction process. Hence, the primary objective of our study is to investigate the mechanistic aspects of this complex reaction system. The CO_2_+H_2_O reaction is particularly challenging due to its thermodynamic constraints, and there is a lack of detailed studies providing clear mechanistic insights into this process. In this work, we aimed to address this gap by investigating the reaction mechanism in the context of varying metal–support interactions. To achieve this, we selected a range of catalyst supports that exhibit varying metal–support interactions, enabling us to understand the dominant reaction pathways. The present work investigates and compares the performance of four different catalysts for the NTP-catalytic conversion of CO_2_ and H_2_O, providing valuable insights into their behavior under plasma conditions. Supported Cu-based catalysts with four different supports (γ-Al_2_O_3_, ZSM5, CeO_2_, and TiO_2_) were applied to the NTP-activated CO_2_+H_2_O reaction. A comparison of the plasma-catalytic activities of Ni/γ-Al_2_O_3_ and Cu/γ-Al_2_O_3_ is also presented. Mechanistic insights into the reaction processes are obtained using *in situ* infrared spectroscopy and catalyst characterization.

## 2 Materials and Methods

The gases used for this experiment are H_2_ (99.98% purity), CO_2_ (99.98% purity), and Ar (99.98% purity). All the gases used in this study were supplied by BOC Ltd.

### 2.1 Catalyst Preparation and Characterization

Copper(II) nitrate trihydrate, nickel(II) nitrate hexahydrate, cerium(III) nitrate hexahydrate, and γ-Al_2_O_3_ were purchased from Sigma-Aldrich and used without any further purification. ZSM5 and TiO_2_ (P25) were purchased from Zeolyst International and Evonik, respectively. CeO_2_ was prepared by thermal decomposition of Ce(NO_3_)_3_·6H_2_O by increasing the temperature from room temperature to 350 °C at a heating rate of a ramp rate of 1 °C/min before maintaining the temperature for 2 h in flowing air (150 mL/min).

Supported Ni (with the theoretical metal loading of 15 wt %) and Cu catalysts (with the theoretical metal loading of 10 wt %) were prepared by the incipient wetness method. The metal precursor solutions were prepared by dissolving each metal nitrate salt in an amount of water sufficient to fill the pores of the support. The support powders (γ-Al_2_O_3_, ZSM5, CeO_2_, and TiO_2_) were first calcined at 400 °C for 5 h and then, after cooling to room temperature, were added to the aqueous nitrate solution and stirred until thoroughly mixed. After 3 h of stirring at room temperature, the resulting mixture was dried overnight at 80 °C. Finally, the dried samples were calcined at 500 °C for 4 h in a muffle furnace. The obtained dry solid was subsequently heated to room temperature at 5 °C/min and reduced in pure H_2_ (100 mL/min) at 400 °C for 2 h.

The structure of the synthesized catalysts was analyzed by powder X-ray diffraction (XRD) using a PANaytical X’Pert PRO diffractometer using Cu K_α1_ radiation at 40 kV, 40 mA. N_2_ physisorption analysis of the prepared catalysts was carried out at −196 °C using a Micromeritics 3Flex Surface Characterization Analyzer. Prior to N_2_ physisorption measurements, the samples (∼100 mg) were degassed at 200 °C under a vacuum overnight. The Brunauer–Emmett–Teller (BET) method was used to determine the specific surface area of catalysts. Temperature-programmed reduction (H_2_-TPR) was performed using a Microtrac BELCAT II equipped with a thermal conductivity detector (TCD). For H_2_-TPR analysis, the sample (∼30 mg) was pretreated in flowing Ar (60 mL/min) at 250 °C for 1 h and then cooled to room temperature under the same flow rate of Ar. The TPR profile was recorded between room temperature and 800 °C at a constant ramp rate of 10 °C/min in 5 vol % H_2_/Ar flowed at 60 mL/min. Temperature-programmed desorption of CO_2_ (CO_2_-TPD) was monitored using the HPR20 Hiden Analytical Mass spectrometer. For CO_2_-TPD, ∼50 mg of sample was loaded and pretreated under flowing Ar at 300 °C for 1 h. After cooling to room temperature, a flow of 10% CO_2_/Ar (at 50 mL/min) was introduced for 1.5 h, followed by a subsequent purge with Ar (50 mL/min) for 1 h to remove the gas phase and physiosorbed CO_2_. The CO_2_-TPD was performed by raising the temperature from room temperature to 800 °C under an Ar flow (50 mL/min) with a temperature ramp of 10 °C/min. For CO-TPD, ∼50 mg of sample was loaded and pretreated under flowing 20 vol % H_2_/He at 400 °C for 2 h. After He purging and cooling to 40 °C, a flow of 10% CO/He (at 50 mL/min) was introduced for 2 h, followed by a subsequent purge with He (50 mL/min) for 1 h to remove the gas phase and physiosorbed CO. The CO-TPD was performed by raising the temperature from room temperature to 800 °C under a He flow (50 mL/min) with a temperature ramp of 10 °C/min. The morphology of the samples was investigated by high-angle annular dark field scanning transmission electron microscopy (HAADF-STEM) using an FEI Titan G2 STEM operated at 200 kV. Energy-dispersive X-ray spectroscopy (EDS) was also carried out using the FEI Titan G2 STEM’s SuperX EDS system (collection solid angle 0.7 srad). X-ray photoelectron spectra (XPS) were recorded on a Kratos AXIS Ultra DLD apparatus with a monochromated Al K_α_ radiation X-ray source, a charge neutralizer, and a hemispherical electron energy analyzer with a pass energy of 160 eV. The binding energies (B.E) were calibrated to the adventitious C 1s peak at 284.8 eV. *In situ* optical emission spectra (OES) of NTP-assisted CO_2_ and H_2_O reactions were detected by an optical spectrometer (USB2000+, Ocean Optics) with a wavelength range from 200 to 900 nm with the exposure time of 500 ms.

### 2.2 Nonthermal Plasma (NTP) Reactions

Figure S1 presents a schematic diagram of the experimental setup used for NTP-catalytic CO_2_–H_2_O reactions. The NTP catalysis was performed in a dielectric barrier discharge (DBD) reactor at atmospheric pressure and without any heating source. The DBD plasma reactor consisted of a cylindrical quartz tube of 6 mm O.D. and 4 mm I.D, where a tungsten wire having a 0.5 mm O.D. was placed inside along the axis of the quartz tube and which acted as the ground electrode. An aluminum foil sheet wrapped around the outer surface of the quartz tube served as the high-voltage power electrode (HV electrode). The discharge gap and the discharge length of the reactor were 1.75 and 15 mm, respectively. An oscilloscope (Tektronix TBS1072B), connected to the reactor through a high-voltage Tektronix, P6015 probe was used to monitor the electrical parameters of the NTP. The DBD plasma was ignited by using an alternating current (AC) high-voltage power supply (Info Unlimited, U.S., PVM500-2500). During each experiment, the discharge zone inside the reactor was packed with ∼80 mg of catalyst (pelletized and sieved to a particle size range of 250–425 μm) and the catalyst was held in place between quartz wool plugs. Prior to the catalytic testing, the as-synthesized catalysts were reduced *in situ* under NTP conditions using 10 vol % H_2_/Ar (peak–peak voltage = 10 kV, frequency = 27 kHz, flow rate = 100 mL/min). The feed gases were controlled by individual mass flow controllers (MFCs, Bronkhorst, F-201CV-500-RAD-11-V). For the reaction, the feed was 2 vol % CO_2_, 2–10 vol % H_2_O (when added), and an Ar balance maintaining the total flow rate of 100 mL/min. Water vapor was introduced by passing Ar through a custom-made water saturator whose temperature was controlled by using a Grant GT120 thermostatic bath. Water condensation was prevented by heat tracing the gas lines before and after the reactor. The applied voltage was varied from 8 to 12 kV (pk–pk) corresponding to the specific input energy (SIE) values of 5.7–13.2 J/mL, at a constant frequency of 27 kHz during each set of experiments. Electromagnetic shielding was implemented all over the rig to reduce noise and any kind of interference signals from external equipment. To ensure accurate average power and specific energy input values, we applied signal averaging during data processing using our custom-programmed online LabView software. The gas exiting the reactor was analyzed by a two-channel online gas chromatograph (GC) equipped with a packed column (HaysepDB), a TCD, and a flame ionization detector (FID) fitted with a methanizer. The GC detects a wide range of gas concentration with TCD identifying H_2_, O_2_, CO, and CO_2_ down to ∼100 ppm, while FID detects CH_4_, CO, CO, and other hydrocarbons down to ∼10 ppm. During the experiment, the concentrations of CO, CO_2_, and CH_4_ were determined from GC-FID data, while H_2_ and O_2_ were quantified using GC-TCD data. The GC was calibrated with five different gas concentrations ranging from 500 to 8000 ppm. For methane, the range was varied from 200 to 1000 ppm considering its low concentration formed during this experiment. Measurement consistency was verified by injecting certified gas standards at least five times under identical conditions, yielding consistent retention times and peak areas. Any change in the gas flow before and after the reaction was monitored using a bubble flow meter. For each experiment, three samples of gas products were injected into the GC and analyzed under steady-state conditions. Control experiments using the empty reactor (plasma-only) and the reactor with the catalyst packing were performed under the same conditions. The stability test of the plasma-catalytic system was conducted by evaluating its activity over time under steady-state plasma conditions. After the initial test, the spent catalysts were exposed to an Ar flow overnight. The next day, they underwent plasma pretreatment before being retested in the CO_2_ + H_2_O experiment under the same conditions.

For the activity evaluation, the following equations were used to define the reactant conversion, products yields, and C, H, and O balance.
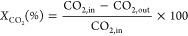
2
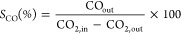
3
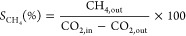
4
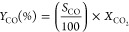
5
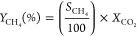
6
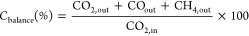
7
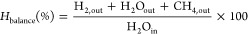
8
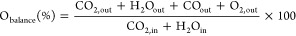
9where *X* is the conversion, *S* is the selectivity, *Y* is the yield, and *C* is the carbon balance.

It should be noted that the carbon balance measured for all of the plasma reactions was found to be in the range of 99–100%, irrespective of the conditions applied.

### 2.3 *In Situ* DRIFTS-MS Characterization for Plasma-Catalytic CO_2_–H_2_O Conversion

The experimental setup for the *in situ* NTP-DRIFTS-MS experiment has been described in detail elsewhere.^30^ It consisted of a custom-built plasma-infrared (IR)–mass spectrometer (MS)-coupled system, where *in situ* DRIFTS measurements were performed using a Bruker Vertex 70 FTIR spectrometer equipped with a liquid nitrogen-cooled detector. The outlet of the DRIFTS cell was connected to a Hidden Analytical HPR20 mass spectrometer via a heated capillary. A type K thermocouple placed inside a quartz capillary tube was served as the high-voltage electrode, inserted underneath the DRIFTS cell, whereas the heating wire was used as a ground electrode, which was wrapped around the sample crucible. The catalyst was placed in a crucible within the DRIFTS cell. Upon applying voltage, the plasma discharge directly interacted with the catalyst bed, similar to the DBD plasma plug-flow reactor used in catalytic testing. The IR beam was focused on the center where plasma discharge was in direct contact with the catalyst surface. The catalyst was loaded into the IR cell and pretreated in a 10% H_2_/Ar flow (50 mL/min) under the plasma (applied pk–pk voltage: 10 kV, frequency: 27 kHz) for 30 min. Then, the reactant gas mixture (2 vol % CO_2_, 2 vol % H_2_O, and balance Ar) was introduced into the cell to initiate the reaction. A constant peak-to-peak voltage of 10 kV at a frequency of 27 kHz was set to avoid arcing between the electrodes. The IR spectra were recorded every 60 s with a resolution of 4 cm^–1^ and analyzed by the OPUS software.

## 3 Results and Discussion

### 3.1 Effect of H_2_O Vapor Content and SIE

**Figure 1:**
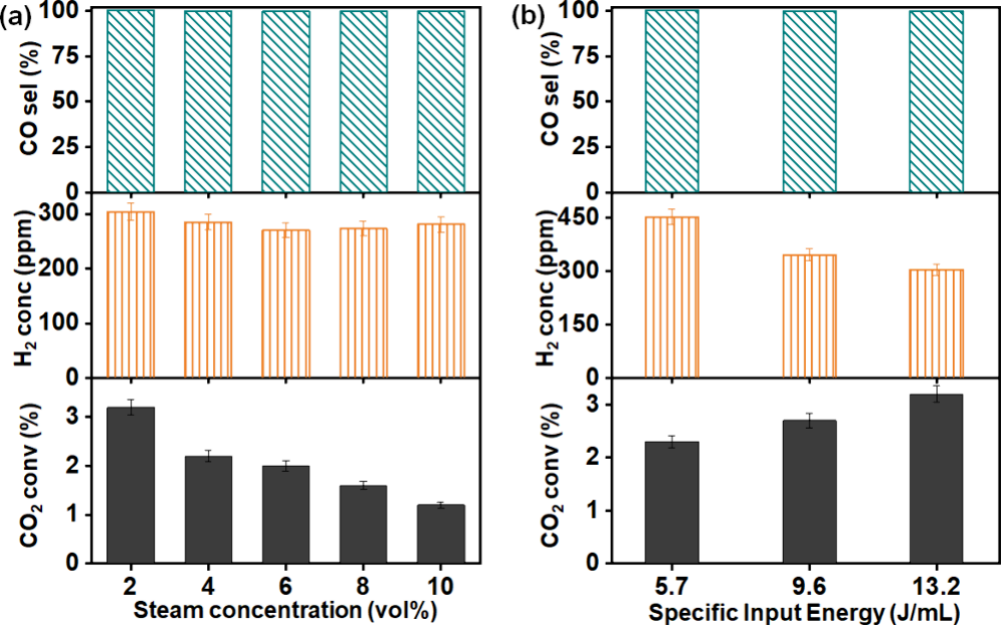
Performance of plasma-only activated CO_2_ and H_2_O conversion as a function of (a) steam concentration and (b) specific input energy in a catalyst-free empty DBD reactor system (reaction condition: total flow rate: 100 mL/min; frequency: 27 kHz).

The effect of water vapor content was studied for the reaction with CO_2_ in an empty DBD plasma reactor (plasma-only). Figure 1a shows that, at a fixed SIE of 13.2 J/mL, the CO_2_ conversion decreases gradually from 3.2 to 1.2% with increasing water content from 2 to 10 vol %. This observation is consistent with the study of Ma et al.^28^ The main products obtained were H_2_ and CO with no significant change observed in the H_2_ concentration with an increasing water concentration above 2%. The CO selectivity was found to be constant and was close to 100% for all the reactant feed compositions used. The decrease in CO_2_ conversion with increasing water concentration may be associated with (i) the water vapor reducing the microdischarges present^28^ and hence reducing the discharge density or (ii) an increasing concentration of hydroxyl radicals (HO•), which react with CO to convert it back to CO_2_. Similarly, changing the total flow rate from 100 to 50 mL/min had an insignificant effect on the CO_2_ conversion, CO selectivity, and H_2_ formation in the case of the blank NTP reactor system (as shown in Table S1).

The effect of SIE was also investigated using the 1:1 molar ratio of the CO_2_–H_2_O feed and is shown in Figure 1b. On increasing the SIE from 5.7 to 13.2 J/mL, the CO_2_ conversion was found to increase while maintaining 100% CO selectivity. Conversely, the H_2_ concentration decreased gradually (Figure 1b), which is likely to be associated with the hydrogen being formed but reacting with the increasing concentrations of oxygen from the CO_2_ conversion as the voltage is increased reforming water.

### 3.2 Plasma Catalysis

**Figure 2:**
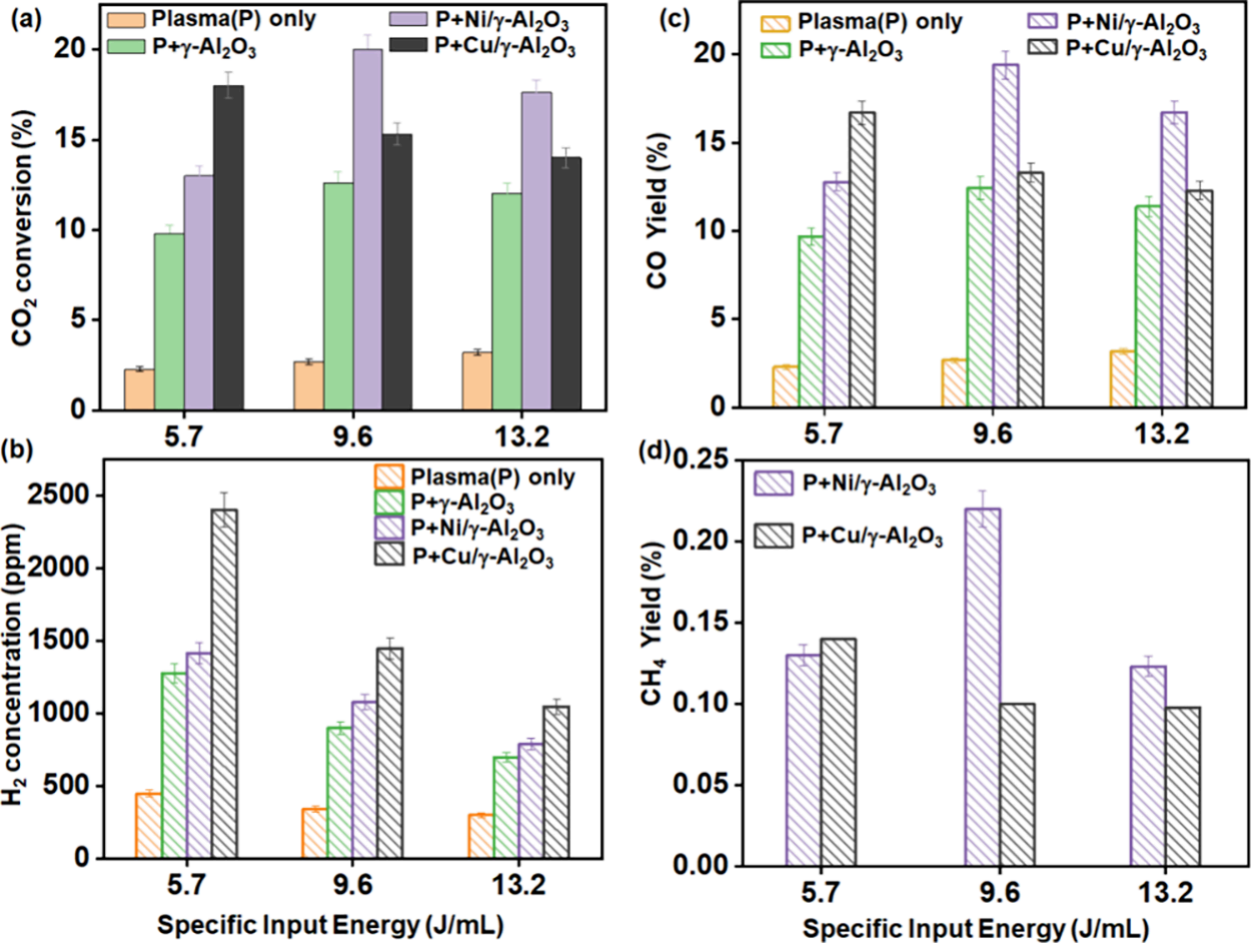
Effect of specific input energy on CO_2_ conversion and the products yield during plasma-catalytic CO_2_ and H_2_O reaction under γ-Al_2_O_3_ support and Ni/γ-Al_2_O_3_ and Cu/γ-Al_2_O_3_ catalysts while comparing the same with plasma-only DBD system (reaction condition: total flow rate: 100 mL/min, CO_2_:H_2_O = 1:1, each 2 vol %).

Figure 2 compares the activity of plasma-only reaction of CO_2_–H_2_O with that in the presence of γ-Al_2_O_3_, Ni/γ-Al_2_O_3_, and Cu/γ-Al_2_O_3_ as a function of the SIE. Unlike the plasma-only condition, the presence of the support/catalyst packing leads to different trends in CO_2_ conversion and product formation with SIE; however, a similar trend in H_2_ formation is observed. For the plasma-only reaction, only 3.2% CO_2_ conversion was achieved at an SIE of 13.2 J/mL (12 kV) resulting in the formation of CO. Similarly, the NTP system with γ-Al_2_O_3_ packing is only selective to the formation of H_2_ and CO with a maximum CO_2_ conversion of 12.6% at 9.6 J/mL (10 kV) (Figure 2). Both Ni/γ-Al_2_O_3_ and Cu/γ-Al_2_O_3_ catalysts show a significant increase in the CO_2_ conversion and CO yield at all SIEs compared with the γ-Al_2_O_3_ support (Figure 2a,c). Interestingly, the presence of Cu and Ni also promoted the formation of small amounts of methane, indicating a surface-catalyzed reaction of CO or CO_2_ with H_2_. The highest H_2_ concentration of 2400 ppm was achieved over the Cu/γ-Al_2_O_3_ catalyst at a CO_2_ conversion of 18% and a SIE of 5.7 J/mL (Figure 2b). The NTP-activated Ni/γ-Al_2_O_3_ catalyst was found to have a maximum CO_2_ conversion of 19.4% and a methane yield of 0.2% at 9.6 J/mL while at the same condition Cu/γ-Al_2_O_3_ exhibited only a 0.1% methane yield (Figure 2d). The increase in H_2_ formation over the catalyst is thought to be due to adsorption of H_2_O on the high surface area material, which increased the residence time of H_2_O on the surface as compared to the blank reactor promoting H_2_ production. The higher H_2_ evolution from the Ni/γ-Al_2_O_3_ and Cu/γ-Al_2_O_3_ coupled NTP system compared with the NTP+γ-Al_2_O_3_ system indicates the critical role of metal active sites in γ-Al_2_O_3_ support, which could facilitate the water adsorption and dissociation. The drop in the reaction performance for Ni/γ-Al_2_O_3_ at higher SIEs >10 J/mL may be correlated with the competitive back reaction of CO with O_2_ (formed during the conversion of CO_2_) to form CO_2_. Similar effects of an increase in SIE has been shown previously to enhance the electron energy to such extent that it could lead to the CO and O_2_ combination over the CO_2_ dissociation.^29^ In addition, although the CO formed in the reaction can also react with water via the water gas shift (WGS) reaction to form CO_2_ and has been shown previously to be enhanced at higher SIE, the contribution of the WGS reaction is thought to be small as there is a decrease and not an increase in H_2_ at higher SIEs.

**Figure 3:**
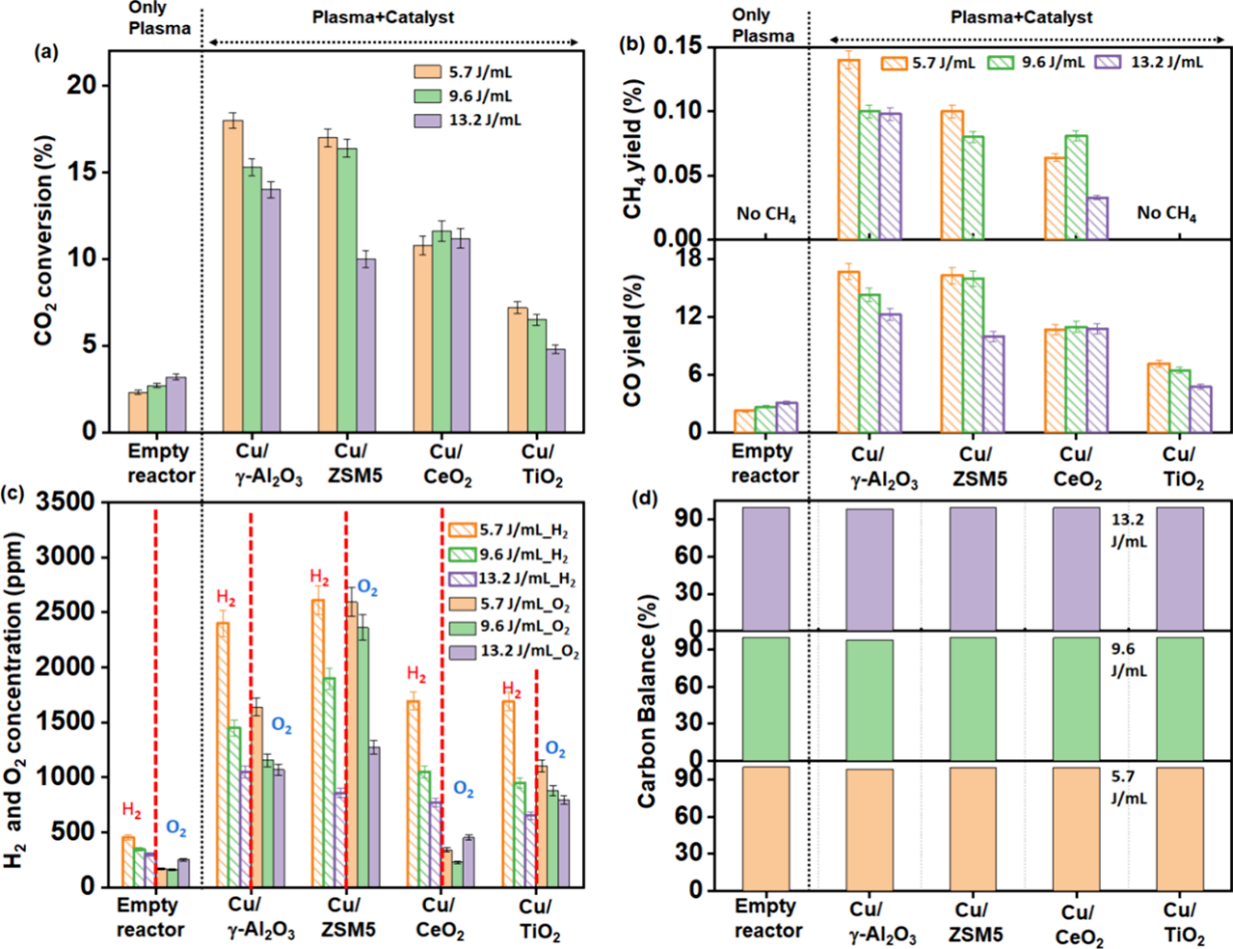
Evaluation of NTP-catalytic reaction of CO_2_ and H_2_O as a function of corresponding specific input energy over various supported Cu catalysts like Cu/γ-Al_2_O_3_, Cu/ZSM5, Cu/CeO_2_, and Cu/TiO_2_ (reaction condition: total flow rate: 100 mL/min, CO_2_:H_2_O = 1:1, each 2 vol %).

As the Cu/γ-Al_2_O_3_ catalyst showed the highest H_2_ yields together with significant CO_2_ conversion to CO, a range of Cu catalysts with different supports were evaluated under the same NTP conditions. The highest H_2_ concentration was obtained over Cu/ZSM5 (2600 ppm) while Cu/γ-Al_2_O_3_ led to the highest CO_2_ conversion at 18% and CH_4_ yield at 0.14% at constant SIE of 5.7 J/mL (Figure 3a–d). Cu/TiO_2_ was the least active toward CO_2_ conversion and did not produce methane but was found to have a significant activity toward H_2_ formation (1690 and 950 ppm at the SIE of 5.7 and 9.6 J/mL, respectively). With the exception of the Cu/CeO_2_ catalyst, 5.7 J/mL was found to be the optimal applied SIE for the other Cu-based catalysts while further increases in SIE resulted in lower CO_2_ conversion and CO yield, again likely as a result of the back reaction of CO with the O_2_ formed in the reaction. The decreasing trend in the H_2_ formation rate with increasing plasma power can be correlated with the fast H• radical abstraction by hydroxyl groups to reform H_2_O.^29^ This process could also be driven by the heat generated from the plasma at higher SIE. The plasma-catalyst-bed temperature was found to be varied with different applied voltages, as measured by the IR temperature sensor. The temperature at the SIE of 5.7 J/mL was found to be in between 60 and 80 °C, which corresponded to the highest H_2_ concentration. However, at higher SIE values of 9.6–13.2 J/mL, the temperature increased to 100–140 °C. In the case of Cu/CeO_2_, there is no significant change in the CO_2_ conversion with respect to the SIE (Figure 3a). This may be explained by the formation of oxygen vacancies on the CeO_2_ surface. Oxygen vacancies create Ce^3+^ sites in the CeO_2_ lattice (as observed in the XPS data below), which can be easily oxidized back to Ce^4+^ by adsorbing and activating CO_2_. This redox cycle plays an important role throughout the reaction with the CeO_2_-based catalyst minimizing the effect of SIE on CO_2_ conversion and CO yield. The H and O balances for all the plasma-catalytic systems were maintained to be in the range of 95–100% (Figure S2).

**Figure 4:**
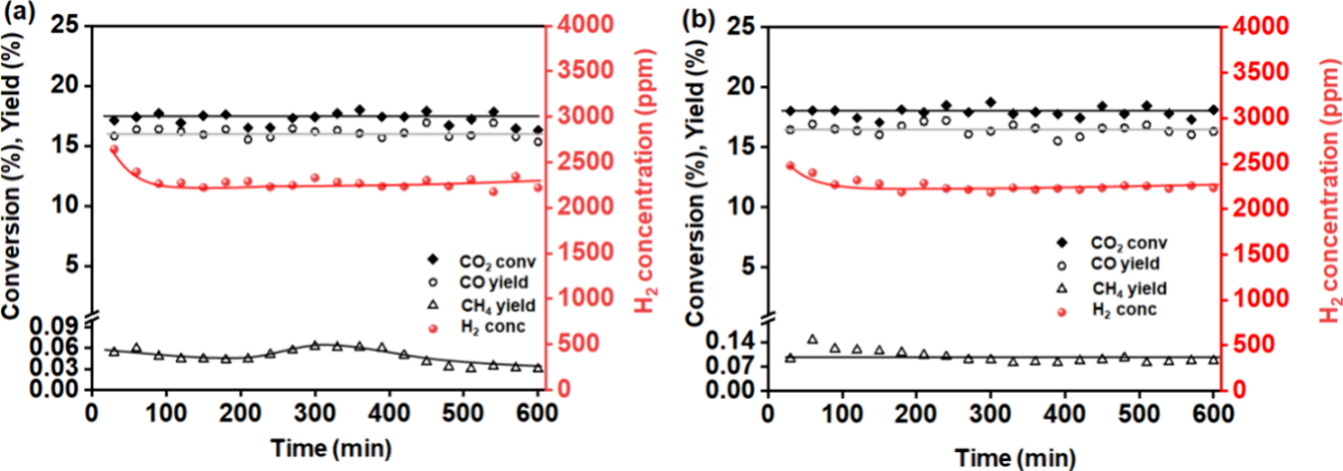
Durability test of the (a) NTP+Cu/ZSM5 and (b) NTP+Cu/γ-Al_2_O_3_ systems under continuous exposure to a CO_2_ and H_2_O (1:1) stream at a flow rate of 100 mL/min, maintaining a constant SIE of 5.7 J/mL.

In order to examine the time-on-stream activity, the Cu/γ-Al_2_O_3_ and Cu/ZSM5 catalysts were examined over a 600 min reaction time. Both catalysts showed good stability at a constant SIE of 5.7 J/mL and a frequency of 27 kHz with a little decrease in H_2_ concentration after an initial period of higher activity over the first 60 min (Figure 4). Notably, the methane concentration was also found to be stable throughout the reaction maintaining a yield of ∼0.1% over Cu/γ-Al_2_O_3_ and <0.1% over Cu/ZSM5.

To explain the catalytic activity on NTP-CO_2_-H_2_O conversion, the catalysts were characterized, and the results were correlated with the reaction data.

### 3.3 Catalyst Characterization

#### 3.3.1 X-ray Diffraction

**Figure 5:**
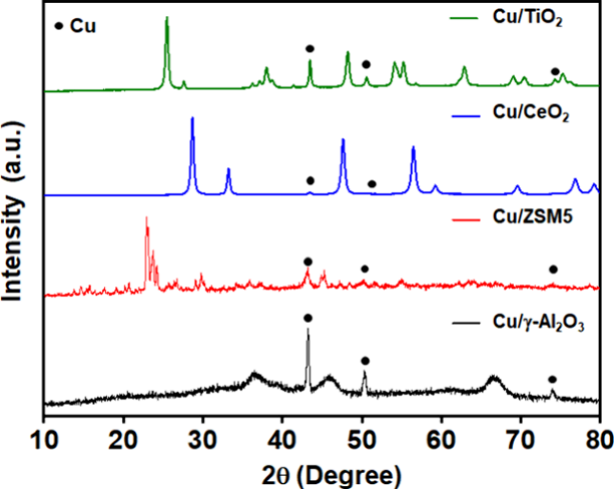
X-ray diffraction patterns of the synthesized catalysts with characteristic Cu planes identified.

From the powder XRD data (Figure 5), all the Cu catalysts only show Cu(0) with characteristic peaks located at 43.3, 50.4, and 74.1° assigned to (111), (200), and (220) planes of Cu, respectively (JCPDS-04-0836). In addition to the Cu peaks, the γ-Al_2_O_3_ support exhibits three major diffraction peaks at 37, 45.9, and 67.0° corresponding to the (311), (400), and (440) planes of the cubic crystalline structure of γ-Al_2_O_3_ (JCPDS reference No. 00-010-0425). Cu/ZSM5 also shows characteristic diffraction peaks at 23.1, 23.9, and 24.2°, which are well-defined superstructure reflections of the ZSM5 zeolite.^31^ The XRD pattern of Cu/TiO_2_ shows sharp crystalline TiO_2_ peaks at 25.3, 37.8, 48.1, 53.9, 55.1, 62.8, 68.8, and 75.2° corresponding to the (101), (004), (200), (105), (211), (204), (116), and (215) planes of the tetragonal anatase structure of TiO_2_ (JCPDS No. 89-4921). The diffraction pattern of Cu/CeO_2_ is characterized by a distinct series of highly intense and sharply defined reflection peaks located at 28.6, 33.2, 47.5, 56.5, 59.1, 69.6, and 76.8°, corresponding to ceria with a fluorite structure.^32^ The reduced Cu phase in Cu/CeO_2_ is only identifiable from the low-intensity peak at 43.3°, suggesting that it is highly dispersed and exists either with an essentially amorphous character or as very small crystallites.

#### 3.3.2 Hydrogen-Temperature-Programmed Reduction (H_2_-TPR)

**Figure 6:**
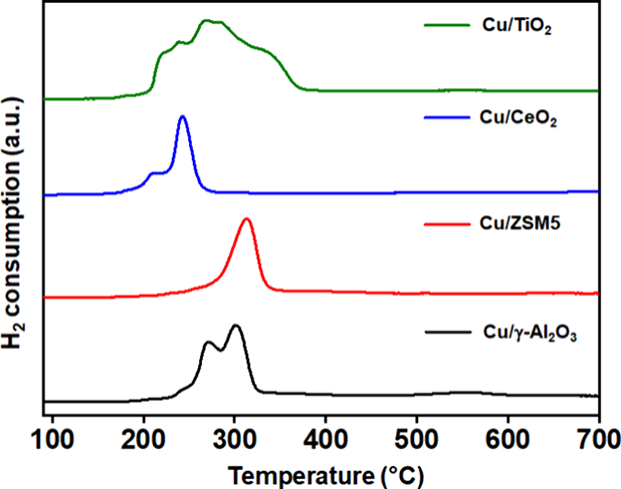
Reducibility of all of the Cu-based catalysts determined by H_2_-TPR.

Figure 6 shows the reducibility of the synthesized Cu-based catalysts measured by H_2_-TPR. Both Cu/γ-Al_2_O_3_ and Cu/CeO_2_ show two distinct reduction peaks. The low-temperature peaks (240–270 °C for Cu/γ-Al_2_O_3_ and 210 °C for Cu/CeO_2_) can be assigned to the reduction of dispersed small CuO particles with a smaller degree of interaction with the support, and the peaks at higher temperatures (300 °C for Cu/γ-Al_2_O_3_ and 242 °C for Cu/CeO_2_) correspond to the reduction of relatively larger CuO particles having a moderate/strong interaction with the support.^33,34^ Cu/TiO_2_ exhibited a broad reduction profile with three peaks in the temperature range of 200 to 340 °C, suggesting the presence of a range of copper–support interactions. The high-temperature peak at 355 °C in Cu/TiO_2_ can also be ascribed to the reduction of bulk crystalline CuO particles. In contrast, the Cu/ZSM5 catalyst only shows one broad reduction peak centered at 315 °C, which can be assigned to the reduction of uniform CuO species strongly interacting with the ZSM5 support. The Cu/CeO_2_ catalyst shows the lowest reduction temperature among all the catalysts, suggesting that CeO_2_ has the weakest interaction with the copper species as compared to other supports. In contrast, ZSM5 and TiO_2_ supports have a comparatively stronger interaction with Cu.^35^

#### 3.3.3 Electron Microscopy

**Figure 7:**
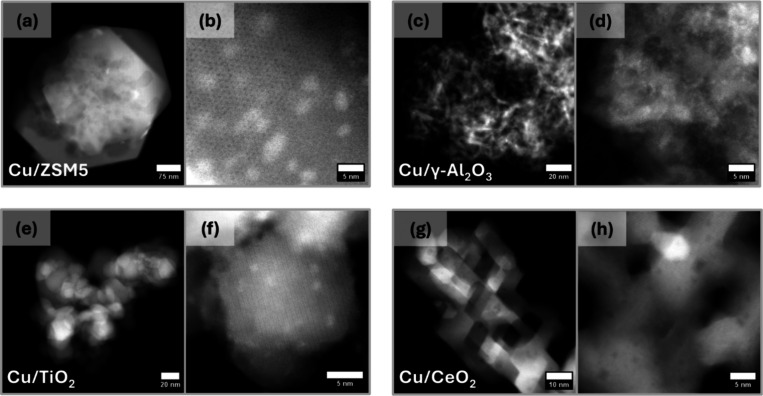
Electron microscopy characterization (*Z*-contrast HAADF-STEM imaging) of the catalysts as synthesized: (a, b) Cu/ZSM5; (c, d) Cu/γ-Al_2_O_3_; (e, f) Cu/TiO_2_; (g, h) Cu/CeO_2_. (a, c, e, g) Lower-magnification images and (b, d, f, h) are higher-magnification images.

HAADF-STEM images were recorded for all the as-synthesized prepared catalysts to understand the Cu dispersion and morphologies. Figure 7 shows that Cu/γ-Al_2_O_3_, Cu/ZSM5, Cu/TiO_2_, and Cu/CeO_2_ all contain small supported nanoparticles. Lower-magnification images (a, c, e, g) show the morphology of each support. Higher-magnification images (b, d, f, h) show the presence of supported nanoparticles with higher intensity (greater density) except in the case of Cu/CeO_2_ (g and h) where the higher atomic number of the support means Cu species will have negligible contrast. The Cu nanoparticles are revealed by STEM EDS mapping (see Figure S3 for the corresponding STEM EDS and size distribution histograms). Particle size measurements give average diameters of 1.0, 1.9, 1.3, and 1.7 nm for Cu/γ-Al_2_O_3_, Cu/ZSM5, Cu/TiO_2_, and Cu/CeO_2_, respectively (see Figure S3 for size distribution histograms). These particles are visible by their greater intensity in the HAADF-STEM images (Figure 7) and/or from STEM EDS elemental Cu maps (Figure S3). All samples contained occasional large Cu particles (diameter >15 nm), although these were relatively very few in number compared to the smaller Cu particles. Consequently, it can be inferred that the smaller, more abundant particles are likely responsible for the observed catalytic activity. As seen from particle size histograms (Figure S3b,g,l), Cu/ZSM5 contains a slightly higher concentration of larger Cu containing particles than either Cu/γ-Al_2_O_3_ or Cu/TiO_2_. Lower-magnification EDS maps of key support elements of all four catalysts and relevant parts of the EDS spectra are also presented in Figure S4.

#### 3.3.4 X-ray Photoelectron Spectroscopy

**Figure 8:**
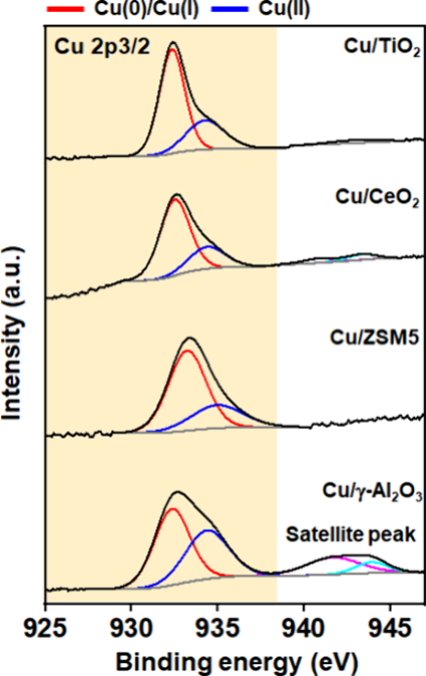
XPS spectra of the deconvoluted Cu 2p_3/2_ peak for all of the fresh catalysts.

XPS analysis was carried out to study the surface composition of all of the catalysts and obtain detailed information about the chemical state of Cu. As illustrated in Figure 8, the main Cu 2p_3/2_ binding energies at 932.3 eV for all the catalysts are attributed to either Cu(I) or Cu(0). An additional peak at a binding energy of 934.4 eV was also observed, which can be attributed to the Cu(II) species.^14,36^ In the case of Cu/γ-Al_2_O_3_, the presence of Cu(II) is supported by the shakeup satellite peaks observed at ∼943 eV (Figure 8). The Al 2p peak is split into contributions from Al_2_O_3_ (Al 2p_3/2_, 74.1 eV) and Al(OH)_3_ (Al 2p_3/2_, 74.9 eV), while the O 1s peak is deconvoluted into lattice O at 530.4 eV and OH at 531.3 eV (Figure S5a and Figure S5b, respectively). For Cu/ZSM5, the Cu 2p_3/2_ peak was observed at 933.2 eV with no satellite peaks present. The absence of satellite peaks in the Cu 2p spectrum is consistent with the presence of only Cu(0) or Cu(I). The O 1s peak can be deconvoluted into two binding energy peaks including 532.7 and 534.9 eV associated with lattice O and surface adsorbed H_2_O, respectively (Figure S6a).^37^ The Si 2p is consistent with the presence of SiO_*x*_ (103.2 eV) and SiO_2_ (103.8 eV) while Al 2p is consistent with Al_2_O_3_ peaks as mentioned above (Figure S6b and Figure S6c, respectively). Cu/TiO_2_ also shows major Cu 2p_3/2_ peaks at 932.3 eV together with O 1s (529.9 and 531.2 eV) and Ti 2p_3/2_ (458.8 eV) peaks, which confirms the presence of Ti(IV) associated with TiO_2_ (Figure S7). In the Cu/CeO_2_ system, the main Cu 2p_3/2_ peak is observed at 932.5 eV. The Ce 3d spectra is characterized by multiple peaks due to the presence of both Ce(III) and Ce(IV) species and their respective satellite peaks with binding energies for the main peaks at 898.0 and 882.1 eV, respectively, for Ce(IV) and Ce(III) 3d_5/2_ features (Figure S8b). The relative content of Ce(III) is 32% as calculated by the ratio of Ce(III) peak area to the total Ce(III) and Ce(IV) peak area, which also indicates the presence of oxygen vacancies in the Cu/CeO_2_ catalyst.^38^ Furthermore, the deconvolution of the O 1s spectra confirms the presence of three oxygen species, namely, lattice oxygen at 529.4 eV, surface adsorbed OH at 531.6 eV, and surface adsorbed H_2_O species at 534.3 eV (Figure S8a).

In summary, the XPS results all showed the presence of oxidized Cu species in all synthesized catalyst materials (from the satellite peaks in Figure 8) with XRD (Figure 5), demonstrating reduced Cu metal also to be present. Deconvolution of the Cu 2p_3/2_ peak provided a measure of the relative distribution of Cu^0^/Cu(I) and Cu(II) species (Table S2). The appearance of satellite peaks at 943 eV in Cu/γ-Al_2_O_3_ strongly suggests the presence of Cu(II) together with the reduced copper species. The observed reduction in signal intensity in the satellite region for the remaining catalysts suggests that Cu(II) is present in significantly lower amounts. This observation may be correlated with the susceptibility of reduced catalysts to oxidation by air during sample transfer, resulting in the generation of copper oxide species on the catalyst surface. Therefore, it can be concluded that the major valence state of Cu exists in Cu/ZSM5 and Cu/CeO_2_, and Cu/TiO_2_ is either Cu(0) or Cu(I), i.e., reduced Cu state while Cu/γ-Al_2_O_3_ has both Cu(I) and Cu(II) with 56.3 and 43.7% relative contribution, respectively. Moreover, all the Cu-based catalysts showed comparable 2p_3/2_ binding energies except for Cu/ZSM5, which has a higher binding energy as compared to others. This is thought to be due to a stronger metal–support interaction between small Cu nanoparticles and ZSM5 as compared with the other oxide supports, which is consistent with the H_2_-TPR results.

To investigate the plasma discharge effects and the resulting active species generated during plasma gas-phase and plasma-catalytic reactions, an *in situ* optical emission spectroscopy (OES) study was conducted. As illustrated in Figure S9, the primary excited species identified during the plasma-activated interaction of CO_2_ and H_2_O include CO_2_, CO, O, OH, and H. A third positive system of CO was detected at 283 and 297 nm along with the presence of CO Angstrom band (*B*^1^ ∑ – *A*^1^ ∏) in the range of 450–560 nm.^39,40^ In addition, an intense OH peak at 309 nm (*A*^2^∑^+^ – *X*^2^ ∏) and a Hα emission line (3*d*^2^*D* – 2*p*^2^*P*) at 656.6 nm^40^ confirm the occurrence of plasma-induced water dissociation. However, it is worth noticing that the relative strength of these active species, especially the H and CO bands, is higher in the spectra of the catalyst-combined plasma system, suggesting an enhanced activation of the reactants in the presence of catalyst. Furthermore, the V–I characteristic plots of the plasma-catalytic systems (Figure S10) revealed a higher frequency of short current pulses compared to the empty reactor system, also suggesting that the catalytic systems could be more conductive to the high-efficiency chemical reaction of CO_2_ and H_2_O. The plasma-catalytic activity data also indicated that the support nature could influence the active species interaction routes. As shown in Figure S11 and Table S3, the BET surface area analysis reveals that Cu/ZSM5 and Cu/γ-Al_2_O_3_ exhibit higher surface areas compared to Cu/CeO_2_ and Cu/TiO_2_. A larger surface area typically correlates with an increased number of accessible active metal sites on the catalyst surface, thereby enhancing interactions with plasma-induced gas-phase reactive species. The average BJH measured pore sizes of the catalysts were found to be in the range of 4.8 to 8.3 nm (Table S3), which are much smaller than the Debye length, suggesting no significant effects of pores on the catalytic performances as the plasma discharge penetration inside these pores will be insignificant.^41^ In general, the higher surface areas of the catalysts led to increased CO_2_ and H_2_O conversion. In addition, the Cu particle size distributions peaked around 1–2 nm (Cu/ZSM5 and Cu/γ-Al_2_O_3_), improving the CO_2_ conversion and CO and H_2_ formation. In addition, the XPS results indicated that the Cu species in a reduced oxidation state is the active Cu oxidation state for CO_2_ and H_2_O conversion. The CO_2_ TPD profiles (Figure S12a) confirm that all the catalysts possess some basic sites that could be beneficial for the CO_2_ chemisorption and activation of CO_2_. These findings also highlight the importance of metal active sites in facilitating dissociative CO_2_ adsorption, which not only increases the probability of CO_2_ dissociation but also elevates its local concentration on the catalyst surface, enhancing interactions with coreactants such as H_2_O. Consequently, this synergistic effect results in higher conversion rates and greater product formation. In terms of the amount of CO_2_ desorbed, the catalysts follow the order of Cu/γ-Al_2_O_3_ > Cu/ZSM5 > Cu/CeO_2_ > Cu/TiO_2_ (Table S3). This trend aligns with the observed CO_2_ conversion efficiency at a SIE of 5.7 J/mL (Figure S12b).

Post plasma reaction catalyst characterization was performed to provide detailed information about the catalyst stability. Figure S13 presents the XRD and XPS analysis of the Cu/γ-Al_2_O_3_ and Cu/ZSM5 catalysts post plasma reaction compared with the fresh samples. The appearance of the distinct Cu_2_O peaks in addition to the metallic Cu peaks in the XRD profile of the spent catalysts clearly suggests that the copper nanoparticles were partially oxidized during the plasma reaction, following 600 min of time-on-stream. The XPS results of spent catalysts further confirm the coexistence of Cu(0)/Cu(I) and Cu(II) species, where the relative concentration of Cu(0)/Cu(I) to Cu(II) species changed from 56.3% (fresh catalyst) to 40.6% (spent catalyst) for Cu/γ-Al_2_O_3_ and from 72% (fresh) to 74% (spent) for Cu/ZSM5. These results suggest that the copper becomes oxidized during the CO_2_+H_2_O plasma reaction by the oxygen generated from the CO_2_ dissociation or H_2_O dissociation. However, the hydrogen formed from H_2_O and CO from CO_2_ dissociation will reduce the catalyst, leading to a dynamic oxidation–reduction process. To determine the stage at which changes in the Cu species occurred during the reaction, the reaction was carried out for durations of 1, 3, 5, and 10 h, followed by characterization of the spent catalysts using XRD. Analysis of the XRD profiles of the spent samples revealed that partial oxidation of the Cu species began after 3 h of reaction for both Cu/γ-Al_2_O_3_ and Cu/ZSM5 catalysts (Figure S14). Beyond 5 h, both catalysts consistently exhibited a partially oxidized Cu(I) state in addition to the Cu(0) state. Although there is a partial oxidation of Cu species, the STEM and elemental mapping analyses of the spent catalysts confirm the comparable results in morphology and uniform distribution of Cu nanoparticles on the support to that of the fresh catalysts (Figure S15). The BET surface areas of Cu/γ-Al_2_O_3_ and Cu/ZSM5 after the time-on-stream reactions were 167 and 301 m^2^/g, respectively, compared with the results from fresh catalysts (172 and 351 m^2^/g, respectively), showing good stability of the catalyst during the reaction.

It is also important to note that the small loss in activity of the spent catalysts could be regained upon H_2_/Ar-plasma pretreatment (Table S4). The plasma pretreatment helps to reduce the partially oxidized Cu nanoparticles back to the reduced Cu state, supporting the proposal that the Cu(0) oxidation state is the most active under these reaction conditions.

### 3.4 *In Situ* DRIFTS-MS

The experimental results suggested that in the plasma-activated CO_2_ conversion with water, in addition to the gas phase reaction, the plasma-assisted surface reaction is significant in determining the CO_2_ conversion and product formation. In order to elucidate the reaction pathways of the NTP-catalytic CO_2_–H_2_O reaction, *in situ* DRIFTS-coupled MS analysis was performed on all of the catalysts studied. Herein, the water was switched into and out of (H_2_O IN and H_2_O OUT) a continuously flowing CO_2_ feed while under plasma discharge, and the surface speciation was compared with the gas phase composition as a function of the water cycles. Three continuous cycles are presented where each cycle consisted of 10 min of H_2_O in and H_2_O out.

The *in situ* DRIFT spectra and the corresponding MS plots for all four catalysts are presented in Figures S16 and S17. Before the NTP is ignited, no CO_2_ conversion is observed in the MS and the DRIFT spectra are characterized by gas phase CO_2_ at 2358 and 2339 cm^–1^. Once the plasma was ignited, the gas phase CO_2_ dissociation to CO and O_2_ were confirmed by the MS and a broad DRIFTS band between 2000 and 2200 cm^–1^ was observed, which corresponds to linearly adsorbed CO species. When water is introduced into the CO_2_+NTP feed, an increase in the CO_2_ and an immediate appearance of the H_2_ together with a decrease in the O_2_ and CO signals in the MS profile were observed. This shows that H_2_O has a negative impact on CO_2_ conversion. The introduction of water also led to significant changes in the DRIFT spectra especially with respect to the CO bands as well as a broad IR band located at 3000–3500 cm^–1^ associated with water adsorption, which increased with the reaction time, as expected.

**Figure 9:**
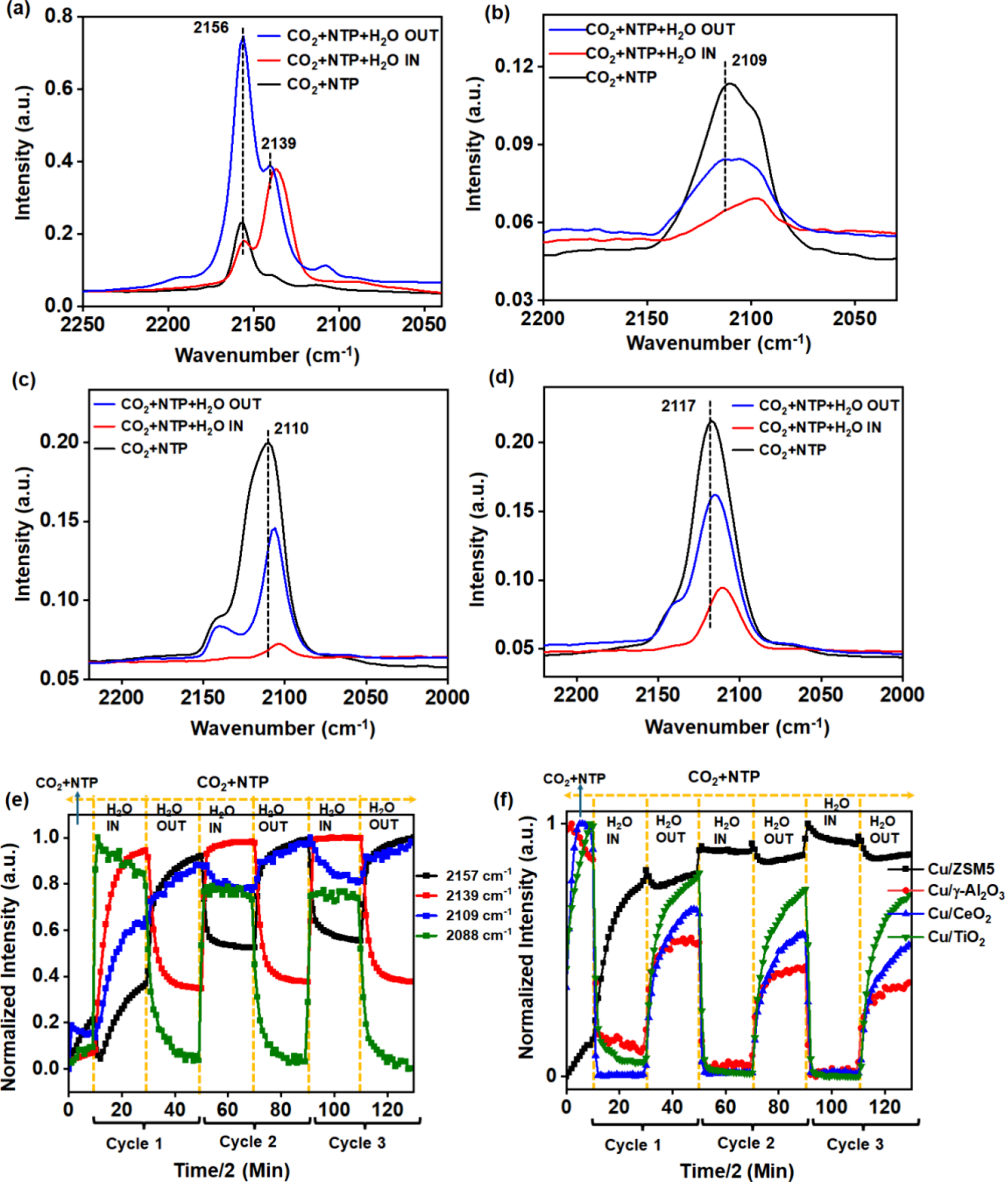
In situ DRIFT spectra of CO species adsorbed on supported Cu catalysts (a) Cu/ZSM5, (b) Cu/γ-Al_2_O_3_, (c) Cu/CeO_2_, and (d) Cu/TiO_2_ under three different conditions: NTP ON with only the CO_2_/Ar feed (2 vol % CO_2_+Ar), NTP ON with the CO_2_/Ar and H_2_O feed (CO_2_:H_2_O = 1:1, 2 vol % each + Ar), and NTP ON with the CO_2_/Ar feed, while H_2_O was taken out, respectively (NTP ON: *V*_P–P_ = 10 kV, frequency = 27.5 kHz). (e) Integrated peak areas of different CO species adsorbed on Cu site at Cu/ZSM5 catalyst. (f) Cumulative CO peak areas for all four catalysts at three consecutive cycles of H_2_O IN and OUT experiments under NTP ON conditions.

For the purpose of this study, which aims to elucidate the interaction pathways of active intermediates, specifically CO in the presence of water, the CO adsorption region of the DRIFT spectra is primarily discussed. For the Cu/ZSM5 catalyst with the plasma ignited, adsorbed CO species were observed at 2157, 2139, 2109, and 2088 cm^–1^, which are attributed to the linearly chemisorbed CO species on both oxidized and reduced copper sites (Figure 9a). The 2157 cm^–1^ peak corresponds to CO adsorption on Cu(II) sites, while the 2139 and 2109 cm^–1^ peaks are associated with Cu(I)–CO adsorption.^42−44^ The CO peak appearing below 2100 cm^–1^ can be ascribed to the CO adsorption on metallic Cu.^44^ In contrast, the major CO band for the other three Cu catalyst surfaces is centered at around 2110 cm^–1^. While there is a shift in wavenumber depending on the catalyst, this may not be associated with a change in oxidation state; for example, Nachal et al. reported that for systems where copper is highly dispersed, Cu(0)–CO species can adsorb at the same frequency as Cu(I)–CO. These two surface species can be differentiated based on their stability with Cu(0)–CO species being more easily removed during flushing or evacuation.^45^ In the present study, the 2110 cm^–1^ band was found to be associated with relatively strongly bound CO to the surface and remained observable even after extinguishing the NTP and flushing with Ar, indicating that it is more likely to be associated with Cu(I)–CO species. The band’s high stability and intensity suggest that these Cu(I)–CO species predominantly populate the surface, although the possibility of minor contributions from the metallic copper carbonyl band cannot be excluded. This proposal is also supported by the post plasma reaction XPS data where Cu(0)/or Cu(I) was found to be the major Cu form existing on the surface. It is also worth noting that the appearance of the oxidized Cu(I) state is primarily due to the NTP+CO_2_ activation, where the O_2_ formed due to CO_2_ decomposition readily oxidizes the Cu, even though the catalyst was pre-reduced.

From Figure 9c and Figure 9d, upon the addition of water (CO_2_+NTP+H_2_O IN), the intensity of the CO band in Cu/CeO_2_ at 2110 cm^–1^ and Cu/TiO_2_ at 2116 cm^–1^, respectively, was found to significantly decrease with a slight shift in the peak position to lower wavenumbers. This indicates the formation of more reducible Cu sites on the surface or the interaction of CO with H_2_O. When water is removed from the feed (CO_2_+NTP+H_2_O OUT condition), the CO peak intensity started to increase again, resembling the initial spectra under the CO_2_+NTP condition. This result suggests that in the presence of water, the adsorbed CO at the Cu(I) site reacts with water under plasma conditions, generating H_2_ via the WGS reaction. This H_2_ is then available to reduce either Cu sites and/or the surface of the CeO_2_ and TiO_2_ support, which explains the CO band shift to lower wavenumbers even after water is removed. When water is excluded from the feed, the O_2_ generated from CO_2_ conversion reoxidizes the Cu, shifting the CO band back to higher wavenumbers but not to its original position. The latter is likely to be because a significant portion of the O_2_ is incorporated into the reducible oxide support rather than fully oxidizing the Cu sites. This is supported by the observation of Ce(III) being present in the XPS data. In the case of the Cu/γ-Al_2_O_3_ catalyst, the CO band bound to the Cu is found at 2110 cm^–1^ (Figure 9b) and is thought to be the active site for this reaction as it shows a reversible change in CO peak position on H_2_O IN and OUT conditions while the NTP is ignited. The CO profile for Cu/ZSM5 differs in that it has bands at 2157 and 2139 cm^–1^, which significantly alter for the CO_2_+NTP+H_2_O IN compared to the CO_2_+NTP+H_2_O OUT (Figure 9a). Upon addition of water (CO_2_+NTP+H_2_O IN), the 2157 cm^–1^ peak is consumed while the 2139 cm^–1^ peak intensity increases. The reverse occurs when water is removed (CO_2_+NTP+H_2_O OUT), again suggesting that the CO bound to the Cu(II) site at 2157 cm^–1^ is the active site for reacting with H_2_O to form H_2_. Examining the integrated CO peak areas (presented in Figure 9e) over the Cu/ZSM5 catalyst shows the appearance of the 2157 cm^–1^ band followed by the 2109 cm^–1^ peak in the absence of water under the CO_2_+NTP condition, suggesting that the O_2_ formed during CO_2_ decomposition leads to CO adsorption at the oxidized Cu site. Upon H_2_O addition, these two peaks initially drop and then slowly increase with time. In addition, the peaks at 2139 and 2088 cm^–1^ continuously increase with this observation found to be most obvious in the second and third cycles. This suggests the possibility of two simultaneous reactions on the Cu/ZSM5 surface:
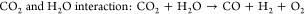
10
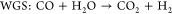
11

The continuous increase in CO peak areas, even when H_2_O is present, indicates that the first reaction (eq 10) is more favorable at the Cu site where CO adsorbed at 2139 cm^–1^. Figure S19 illustrates the initial transition phase from CO_2_+NTP to CO_2_+NTP+H_2_O conditions (cycle 1), highlighting the distinct behavior of CO bands upon the introduction of H_2_O. Upon H_2_O addition, there is a significant initial drop for the bands at 2157 and 2109 cm^–1^, which suggests removal of CO via the WGS reaction (eq 11) at these two sites. At the same time, a steady increase was observed for the bands at 2139 and 2088 cm^–1^ under the same conditions, which implies the continuous reaction between CO_2_ and H_2_O to form CO (eq 10). These changes become more pronounced in cycles 2 and 3 (Figure 9e). Unlike those over the other three catalysts, CO adsorption on the Cu/ZSM5 surface is much stronger and becomes saturated over time. This is also supported by the corresponding CO-TPD data where Cu/ZSM5 was found to have the highest CO coverage with a total amount of 0.319 mmol/g of CO adsorbed followed by Cu/γ-Al_2_O_3_ (0.276 mmol/g), Cu/TiO_2_ (0.135 mmol/g), and Cu/CeO_2_ (0.107 mmol/g). It can also be seen that the high capacity for CO adsorption on Cu/ZSM5 led to varying CO peak heights in Figure 9a compared to those in Figure 9b–d. The high surface area of Cu/ZSM5 might offer multiple sites for CO adsorption, as evident from Figure S18. Figure 9f illustrates the changes in the overall CO peak area (measured from the DRIFT spectra) over time during cyclic H_2_O IN and OUT conditions. In the first H_2_O IN cycle, the decrease in adsorbed CO follows the order Cu/CeO_2_ > Cu/TiO_2_ > Cu/γ-Al_2_O_3_ > Cu/ZSM5, which may also indicate the rate of the WGS reaction to form H_2_. This order does not reflect the order of hydrogen production in the gas phase, and this may be explained by the utilization of the hydrogen formed to reduce the Cu/CeO_2_ and Cu/TiO_2_ catalysts, resulting in lower H_2_ formation than Cu/ZSM5 and Cu/γ-Al_2_O_3_. For Cu/ZSM5, the simultaneous occurrence of both the CO_2_+H_2_O reaction and the WGS reaction may explain the higher concentration of H_2_ formation.

To understand the long-term stability of the CO_2_+H_2_O reaction (presented in Section 3.2, Figure 4), *in situ* DRIFTS-MS was also used to examine the system under a continuous stream of CO_2_ and H_2_O over Cu/ZSM5 and Cu/γ-Al_2_O_3_ catalysts under NTP ON conditions for 100 min. The MS results in Figure S20 show an initial high intensity of the H_2_ signal followed by a decrease, which then stabilized, mirroring the activity test data (Figure 4). For Cu/ZSM5, the trend of the product (CO, H_2_) formation can be correlated with the CO_2_ and H_2_O conversion (inset in Figure S20b). From Figure S20a,b, the initial increase in the H_2_ signal aligns with the initial spike in the H_2_O signal, indicating a high H_2_ concentration resulting from H_2_O conversion. Similarly, the CO_2_ and CO signals exhibit complementary behavior. This suggests the occurrence of the reaction described in eq 10, as previously mentioned. The changes in the CO peak area adsorbed over both oxidized and reduced Cu sites at Cu/ZSM5 are also presented. The 2139 cm^–1^ peak rapidly increases over time, while the 2157 cm^–1^ peak increases at a much slower rate (Figure S20c,d). This suggests that although both reactions (eqs 10 and 11) occur on the Cu/ZSM5 surface, the rate of reaction shown in eq 10 at the Cu(I) site is faster than that of the WGS reaction (eq 11). This difference in rate is responsible for the initial higher H_2_ formation, which continues until the rate of these two reactions (eqs 10 and 11) becomes equal, resulting in a stable run over time. Unlike Cu/ZSM5, in the case of Cu/γ-Al_2_O_3_, the rapid decrease in the CO peak area with time indicates the rapid consumption of CO or reaction of CO with H_2_O to form H_2_ (Figure S20e,f). Hence, it can be suggested that on Cu/γ-Al_2_O_3_, the rate of the WGS reaction (eq 11) is much faster than the rate of the CO_2_+H_2_O reaction (eq 10) to form H_2_.

## Conclusions

NTP-catalytic conversion of CO_2_ and H_2_O is strongly dependent on the nature of the CO adsorption on Cu, which is determined by the oxidation state and the interaction of Cu with the support. The *in situ* DRIFTS characterization indicated that this CO is a key species for the reaction and is formed in the gas phase via NTP-activated CO_2_ dissociation or by the surface-mediated dissociative adsorption of CO_2_. The CO formed and adsorbed on the surface then reacts with H_2_O via the WGS reaction to generate H_2_. In general, reduced copper species promote the reaction; however, the reducibility of the support and the redox behavior of the systems appear to control the outcome of the reaction. For example, while there is an indication that TiO_2_ and CeO_2_ catalysts may promote the WGS reaction and thus should enhance the hydrogen production, the subsequent reduction (and reoxidation with the oxygen formed from the CO_2_ conversion to CO in the plasma) limits the availability of the hydrogen under plug-flow conditions. Therefore, nonreducible oxides but with some sites that can adsorb CO_2_ provide an optimum system under these conditions. It should be noted that it may be possible to engineer the process through membrane separation of H_2_ or capture through organic hydrogen carriers, depending on the reforming activity of the system, to further enhance the hydrogen production. While the DBD plasma has demonstrated promise as an effective setup for such laboratory-scale research particularly for catalyst integration with a better control of residence time at lower flow rates, we recognize that alternative reactor designs with a stable plasma power source, controllable feed flow rates, and optimized catalyst placement could further improve such processes and open new avenues for future research. Overall, the Cu/ZSM5-coupled plasma system resulted in the highest H_2_ yield and a small production of methane with good stability with time-on-stream. Deactivation of the catalyst system was found to be due to oxidation of the copper sites, and these could be regenerated with a reductive treatment. Importantly, the cogeneration of oxidative species, which limits the H_2_ and CO production, as well as hydrocarbons potentially, as well as controlling the stability of the catalyst must be addressed through removal via physical means or reacting them to further make this a practical process.

## Data Availability

Supporting datasets are openly available via the Figshare repository, https://doi.org/10.48420/28740821.

## Supporting Information Available

Experimental setup; effect of flow rate variation on NTP-CO_2_+H_2_O reaction; H and O balance; HAADF-STEM images and the corresponding EDS maps of all the key elements of the fresh catalysts; XPS spectra of all four catalysts and data analysis from the deconvoluted Cu 2p3/2 peak; OES analysis and V–I characterization; BET plot and CO_2_ TPD characterization; table of physiochemical properties of the fresh catalysts; detail of spent catalyst characteristics; NTP-catalytic stability test; CO-TPD data analysis for all four catalysts; and *in situ* DRIFT-MS data for all four catalytic system under NTP ON and H_2_O IN/OUT condition (PDF)
