# Supplementary material for: Enhancing the Reaction of CO2 and H2O Using Catalysts within a Nonthermal Plasma
Source: ACS Catal. 2025 Apr 16;15(9):7053–65. doi: 10.1021/acscatal.5c00747 (PMC12053938; doi:10.1021/acscatal.5c00747)
Supplement: Supplementary file 1 — cs5c00747_si_001.pdf [file cs5c00747_si_001.pdf]

Supporting Information

Enhancing the reaction of CO_2 and H_2O using catalysts within a non-thermal plasma

Piu Chawdhury,[1,*] Sarayute Chansai,[1] Matthew Conway,[2] Joseph Parker,[3] Matthew Lindley,[3] Cristina E. Stere,[1] Meenakshisundaram Sankar,[2] Sarah J. Haigh[3], Ben Dennis-Smither,[4] Sorin V. Filip,[5] Stephen Poulston,[6] Peter Hinde,[7] Christopher Hawkins,[6] Christopher Hardacre[1,*]

[1]*Department of Chemical Engineering and Analytical Science, The University of Manchester, Oxford Road, Manchester M13 9PL, UK*
[2] *Cardiff Catalysis Institute, School of Chemistry, Cardiff University, Maindy Road, Cardiff CF24 4HQ, United Kingdom*
[3]*Department of Materials, The University of Manchester, Manchester, M13 9PL, United Kingdom4*
[4]*Low carbon innovation centre, BP International Ltd, Saltend Chemicals Park, Hull, HU12 8DS, UK*
[5] *BP Technology Centre, Whitchurch Hill, Pangbourne, RG8 7QR*
[6]*Johnson Matthey Technology Centre, Blount's Court, Sonning Common, Reading, RG4 9NH, UK*
[7]*JM Technology Centre, Chilton Site, Belasis Avenue, Billingham, TS23 1LB, UK*

Corresponding Author: c.hardacre@manchester.ac.uk, piu.chawdhury@manchester.ac.uk

List of contents

1. Experimental set-up.
2. Effect of flow rate variation on NTP-CO_2+H_2O reaction.
3. H and O Balance.
4. Electron microscopy characterization: HAADF STEM images recorded before and after EDS mapping with the corresponding EDS maps of all the key elements of the fresh catalysts.
5. XPS spectra of all 4 catalysts.
6. XPS data analysis: The relative distribution of Cu species exhibiting different oxidation states.
7. OES analysis and V-I characterization.
8. Catalyst characterisation including BET plot, CO_2 TPD data and a table of physiochemical properties of the fresh catalysts.
9. Spent catalyst characterisation.
10. NTP-catalytic stability test.

11. *In-situ* DRIFT-MS data for all 4 catalytic system under NTP ON and H_2O IN/OUT condition.

12. CO-TPD data analysis for all 4 catalysts.

13. A deep insight into the *In-situ* DTIFT-MS characterization data for Cu/ZSM5 and Cu/γ-Al_2O_3.

1. Experimental set-up and GC calibration details:

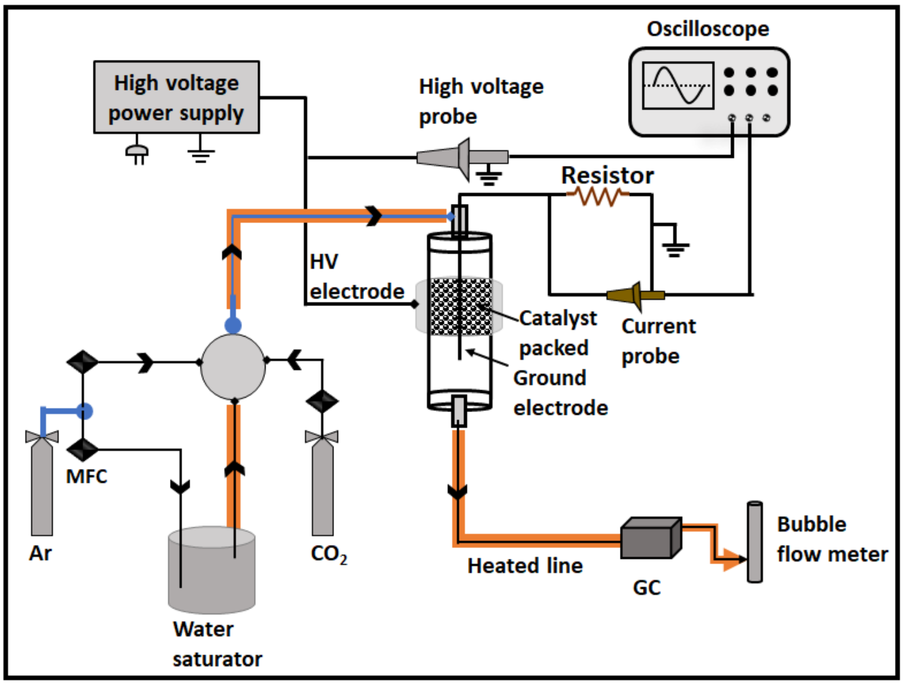

Figure S1. Schematic of the NTP reactor set-up for the conversion of CO_2 and H_2O.

2. Effect of flow rate variation on NTP-CO_2+H_2O reaction:

Table S1: Flow rate variation in blank NTP reactor system

Experimental condition: CO_2:H_2O = 1:1 (2 vol% each) with Ar as balance gas at constant SIE of 13.2 J/mL and a frequency of 27 kHz.

Total flow rate (mL/min)	CO_2 conversion (%)	CO selectivity (%)	H_2 concentration (ppm)
100	3.2	99.9	302
75	3.6	99.2	329
50	3.7	99.0	372

3. H and O Balance:

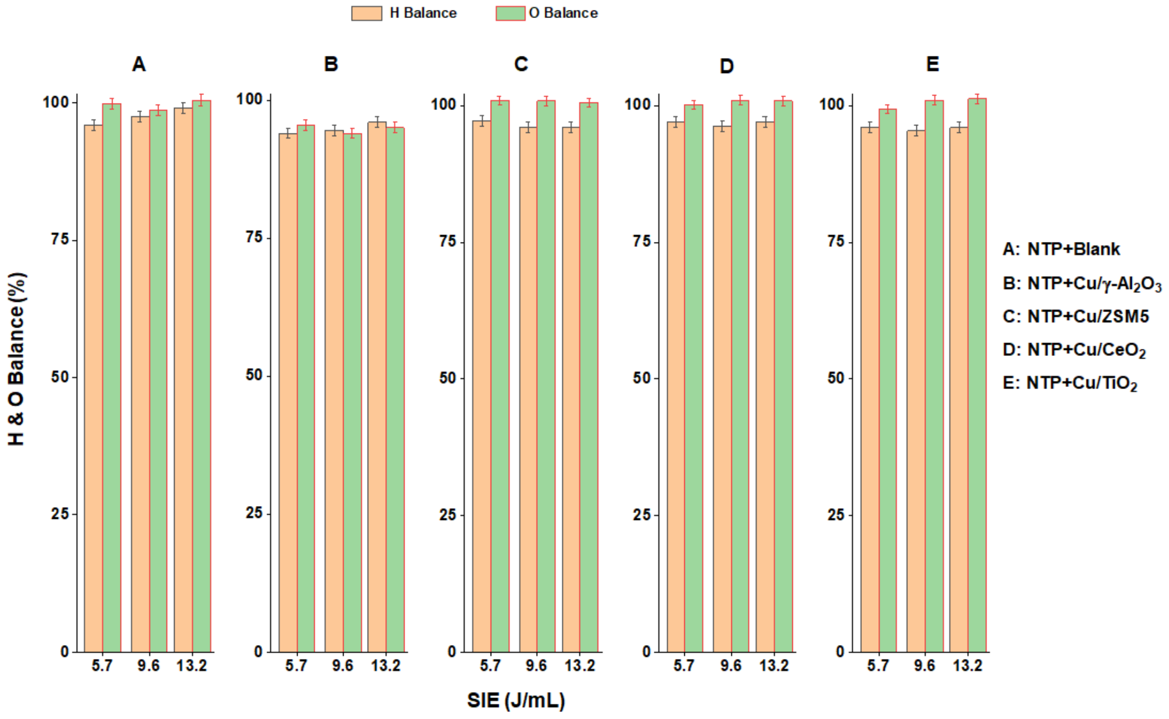

Figure. S2. H and O Balance were calculated for both blank reactor and different catalyst combined NTP reactor systems. (Reaction condition: Total flow rate: 100 mL/min, CO_2:H_2O = 1:1, each 2 vol%)

4. **Electron microscopy characterisation:** HAADF STEM images recorded before and after EDS mapping with the corresponding EDS maps of all the key elements of the fresh catalysts.

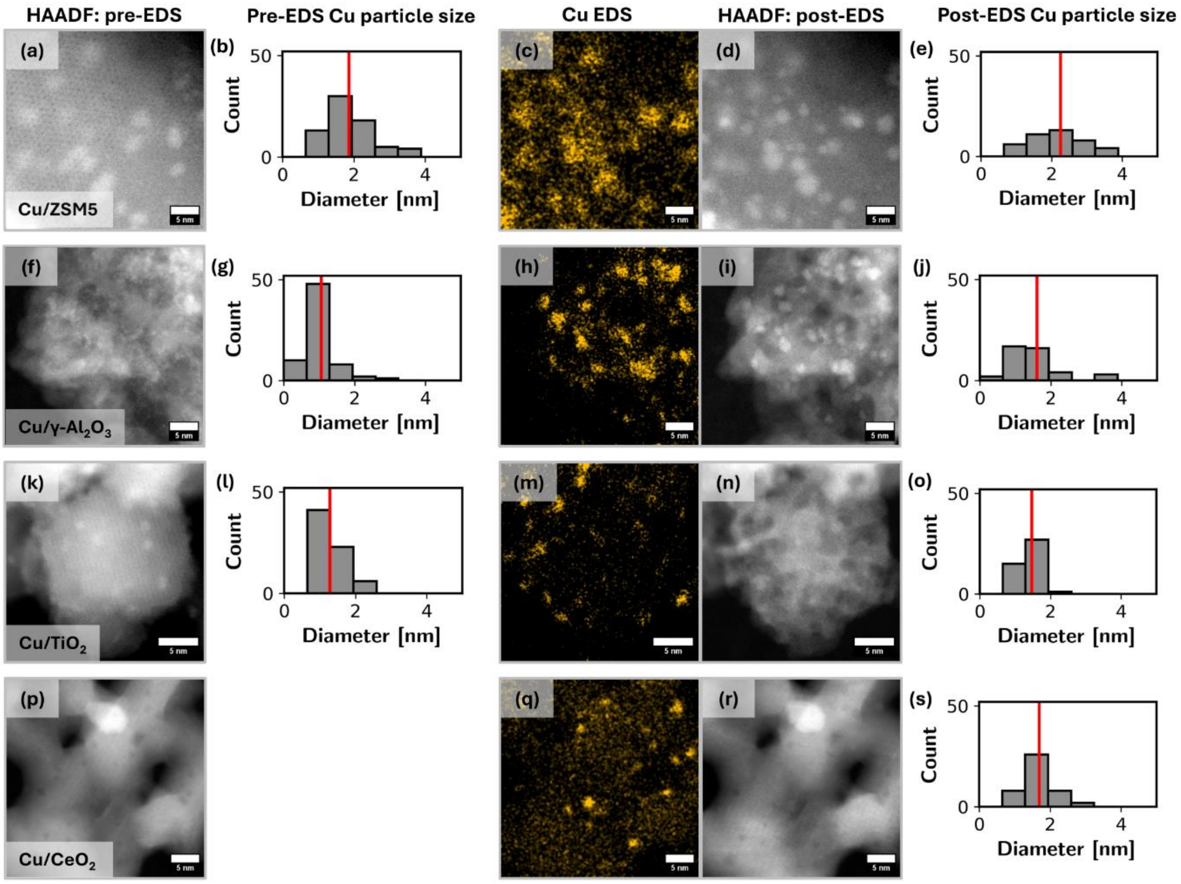

Figure S3. HAADF STEM images, particle size distributions and Cu EDS elemental maps for each catalyst, accounting for significant electron beam damage. (a-e) Cu/ZSM5; (f-j) Cu/γ-Al_2O_3; (k-o) Cu/TiO_2 and (p-s) Cu/CeO_2. The first column (a, f, k & p) shows HAADF STEM images before EDS mapping, with little or no beam damage. Particle size distributions have been produced from these and similar pre-EDS images of each catalyst (b, g & l, with 70 particle measurements each) except for Cu/CeO_2 where there is no Cu/Ce image contrast. Vertical red lines mark mean diameters. EDS maps (c, h, m & q) show Cu-containing nanoparticles. Further HAADF images acquired after EDS (d, i, n & r) suggest significant

movement and agglomeration of nanoparticles due to the electron beam. A second set of particle size distributions (e, j, o & s, with 44 particle measurements each) has been produced from EDS maps and post-EDS images, demonstrating growth due to beam damage. Cu/CeO_2 particle size (s) can only be estimated from EDS maps and is therefore affected by beam damage. Pre- and post-EDS images, as well as the EDS maps themselves, correspond to identical regions in each catalyst.

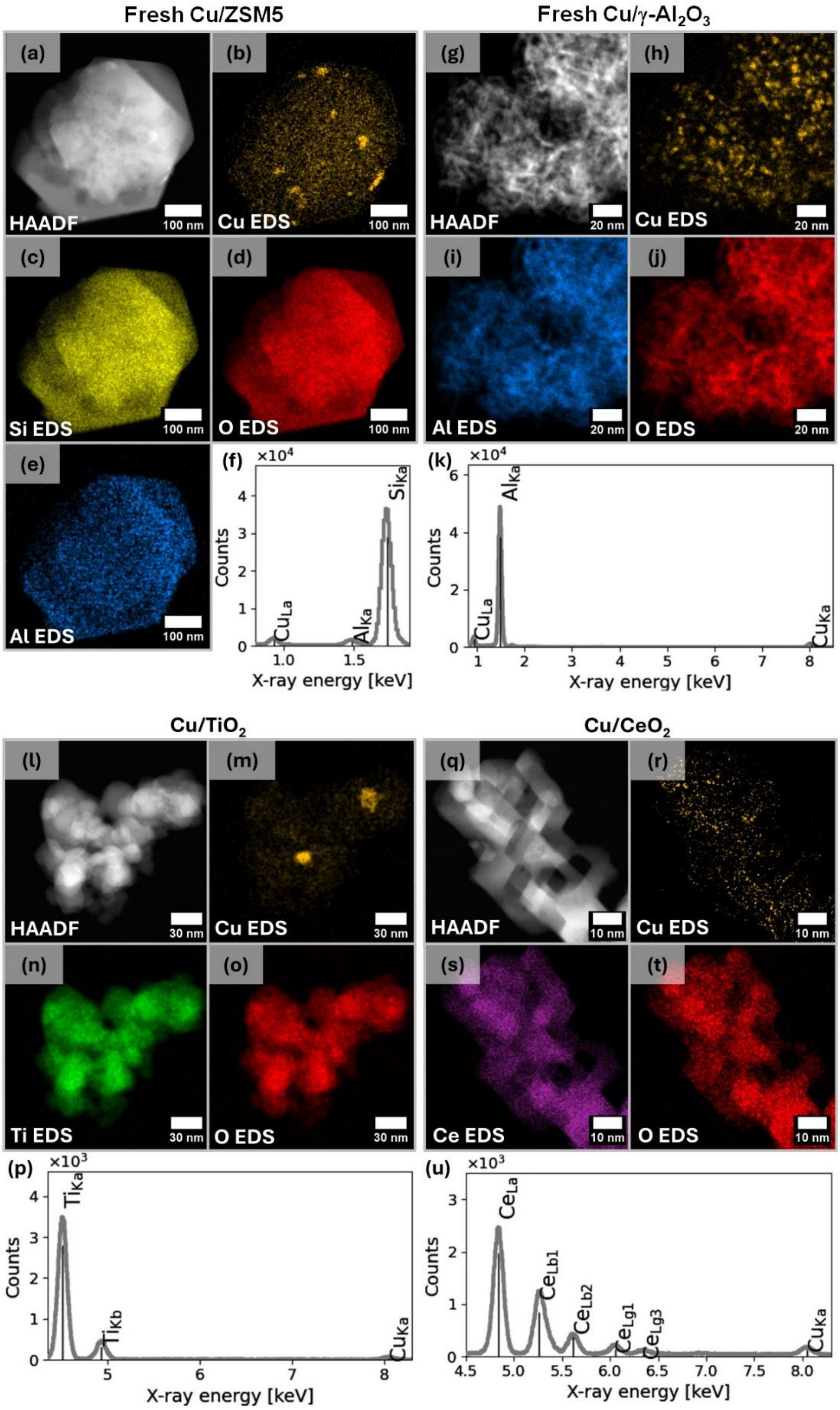

Figure S4. Electron microscopy characterisation of the as synthesised catalysts. (a - f) Cu/ZSM5; (g - k) Cu/γ-Al_2O_3; (l - p) Cu/TiO_2; (q - u) Cu/CeO_2. Where (a, g, l, q) are HAADF-STEM images; (b, h, m, r) are the Cu EDS maps of the are shown in their corresponding

HAADF STEM images; EDS maps of key support elements are presented in (c – e) Cu/ZSM5, (i, j) Cu/γ-Al_2O_3, (n, o) Cu/TiO_2, (s, t) Cu/CeO_2, respectively; (f, k, p, u) are the EDS spectra obtained from the corresponding maps.

5. XPS spectra of the fresh catalysts

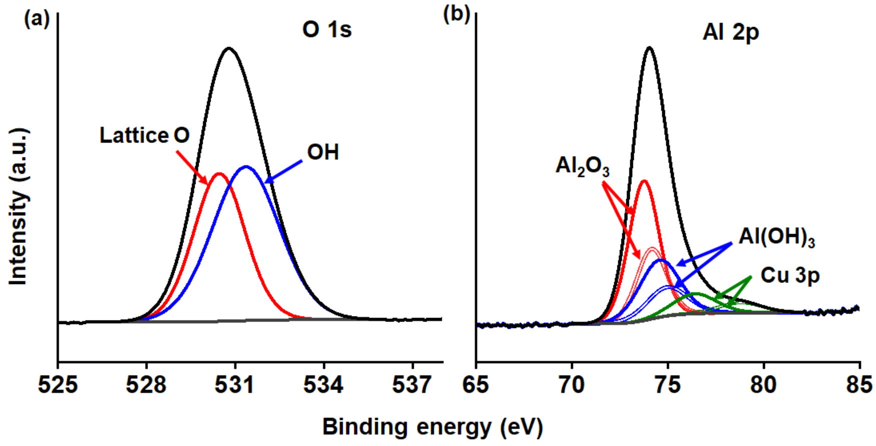

Figure S5. XPS spectra of fresh Cu/γ-Al_2O_3, deconvoluted peaks of (a) O 1s and (b) Al 2p.

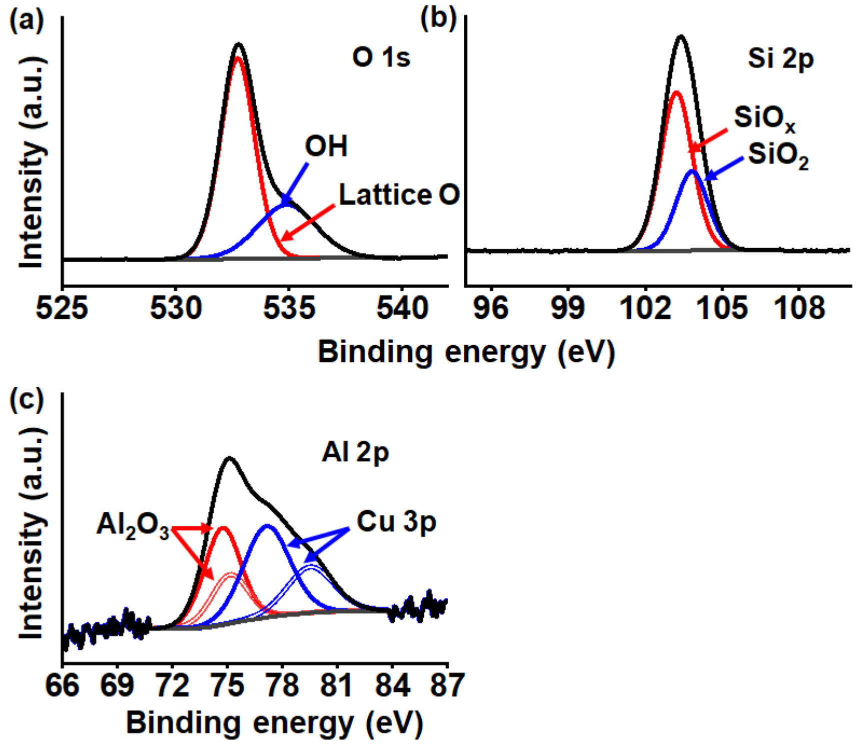

Figure. S6. XPS spectra of fresh Cu/ZSM5, deconvoluted peaks of (a) O 1s (b) Si 2p and (c) Al 2p.

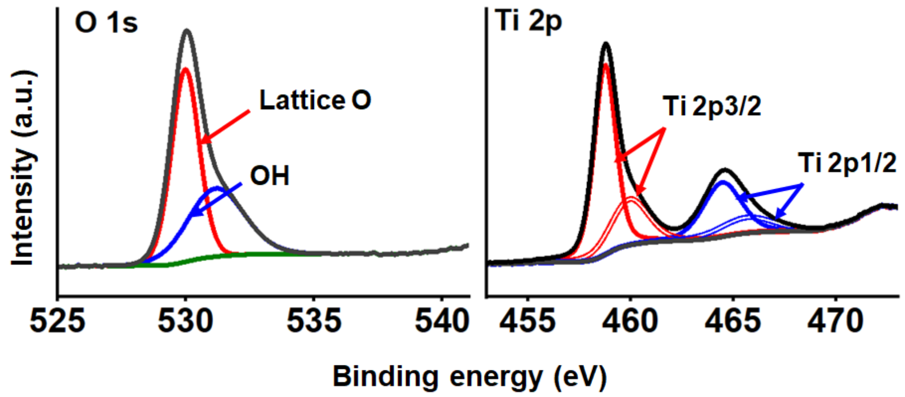

Figure. S7. XPS spectra of fresh Cu/TiO_2, deconvoluted peaks of (a) O 1s and (c) Ti 2p.

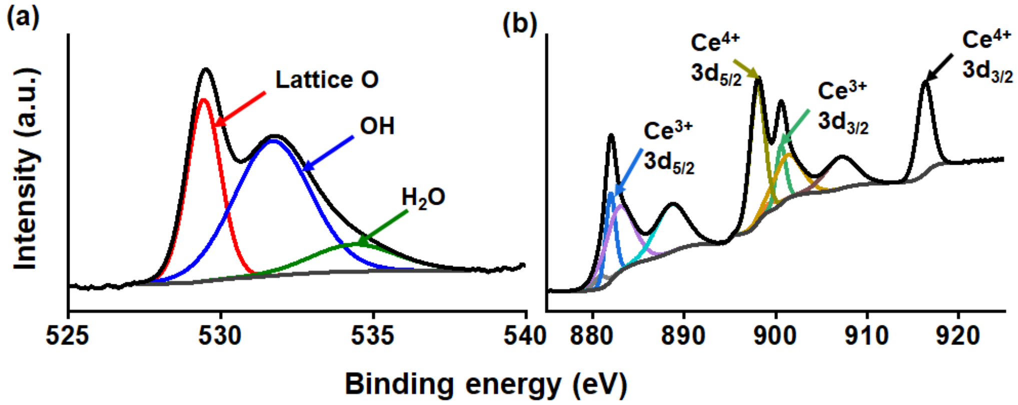

Figure S8. XPS spectra of fresh Cu/CeO_2, deconvoluted peaks of (a) O 1s and (b) Ce 3d.

6. **XPS data analysis:** The relative distribution of Cu species exhibiting different oxidation states.

The relative distribution of Cu(0)/or Cu(I) and Cu(II) values for all the catalysts were calculated from the deconvoluted Cu $2p_{3/2}$ peak.

Table S2: XPS data for the relative distribution of the Cu species exist on the freshly prepared catalysts

Reduced Cu = Cu(0)/or Cu(I) or Cu(0)+Cu(I)

Cu(Total) = Reduced Cu + Cu(II)

**Catalyst**	**Reduced Cu/Cu(Total)]×100/%**
Cu/Al_2O_3	56.3
Cu/ZSM5	72.0
Cu/CeO_2	74.0
Cu/TiO_2	70.0

7. OES Analysis and V-I characteristics

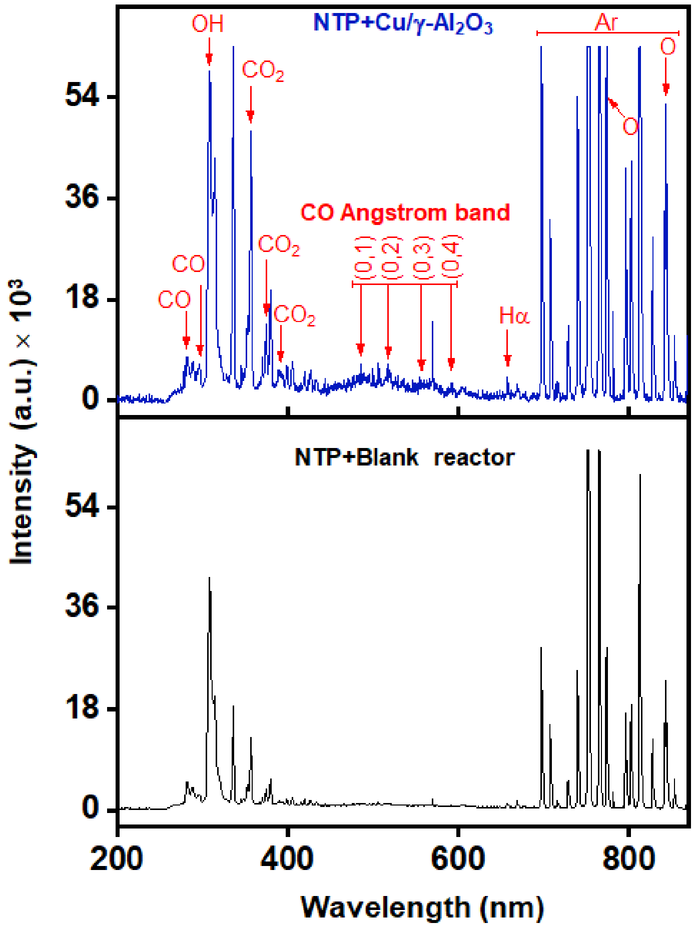

Figure S9. In-situ optical emission spectra in NTP alone (blank reactor) system and NTP-catalyst (Cu/γ-Al_2O_3) system (Exp condition: CO_2:H_2O = 1:1, TFR= 100 mL/min, SIE of 5.7 J/mL, Frequency: 27kHz).

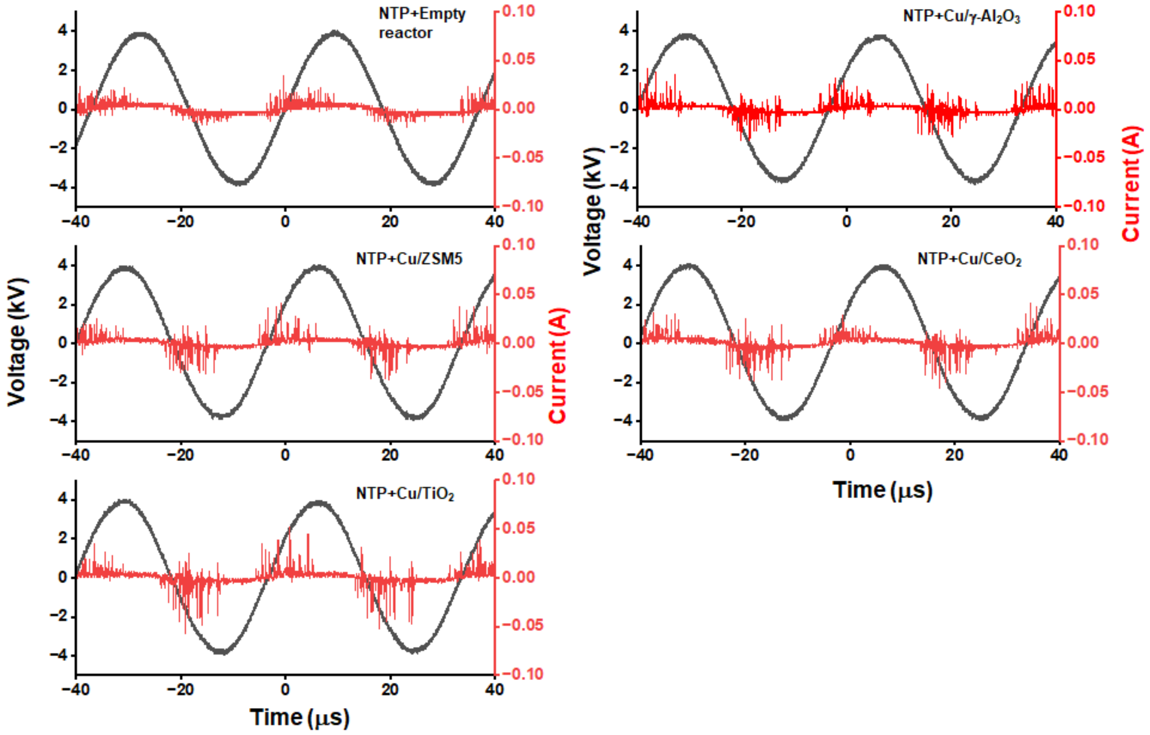

Figure S10. V-I characteristics of NTP+empty reactor system and NTP+catalyst systems (Exp condition: CO_2:H_2O = 1:1, TFR= 100 mL/min, SIE of 5.7 J/mL, Frequency: 27kHz).

8. Catalyst characterization

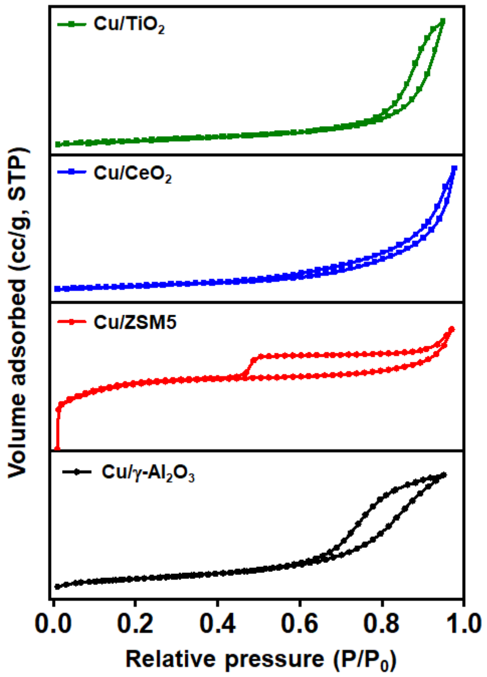

Figure S11. N_2-adsorption-desorption isotherms of the fresh catalysts.

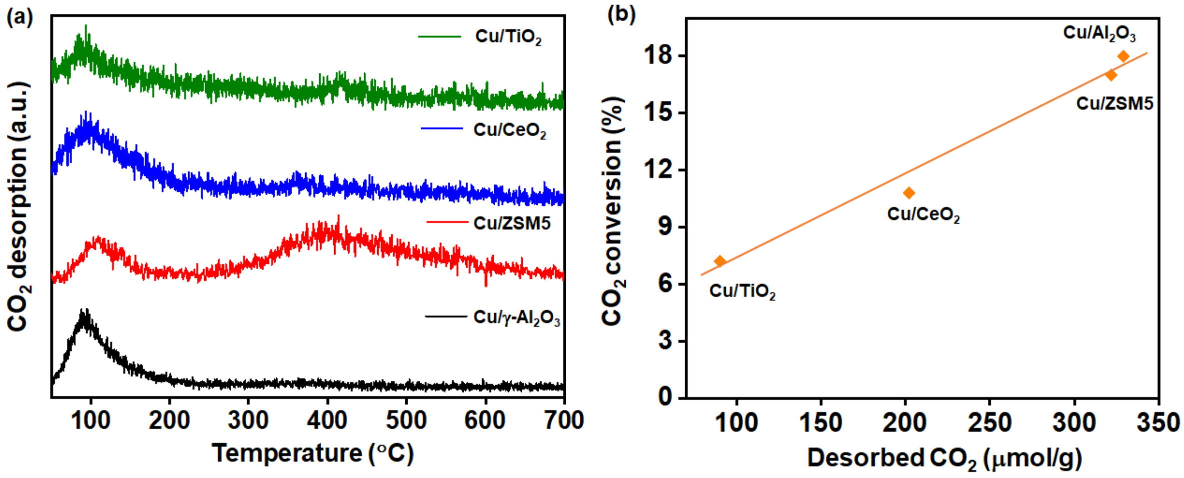

Figure S12. (a) Temperature programmed CO_2 desorption curves for the freshly prepared catalyst, (b) A linear relationship observed between the NTP-catalytic CO_2 conversion and the quantity of CO_2 desorbed from the catalyst surfaces. The amount of desorbed CO_2 from each catalyst surface were measured by their corresponding CO_2 TPD curves.

Table S3: Physiochemical properties of the fresh catalysts.

**Catalyst**	**Specific Surface area (m^2/g)[a]**	**Average pore size (nm)[b]**	**T-Plot micropore area (m^2/g)[c]**	**T-Plot micropore volume (cm^3/g)[c]**	**Desorbed CO_2 amount (µmol/g)[d]**
Cu/γ-Al_2O_3	167.6	8.3	29.49	0.0148	328.8
Cu/ZSM5	351	4.8	253.78	0.130	321.8
Cu/CeO_2	29.8	14.9	9.22	0.0046	202.3
Cu/TiO_2	36.6	14.9	7.07	0.0035	90.1

[a]The specific surface area of the catalysts was calculated using the Brunauer–Emmett–Teller (BET) method.

[b]Average pore size of the catalysts was measured using the Barrett-Joyner-Halenda (BJH) method.

[c]Micropore volume and micropore area were measured using the T-Plot method.

[d]The amount of desorbed CO_2 from the catalyst surface was measured by using the integrating the CO_2 signal from the Mass spectrometer.

9. Spent catalyst characterizations.

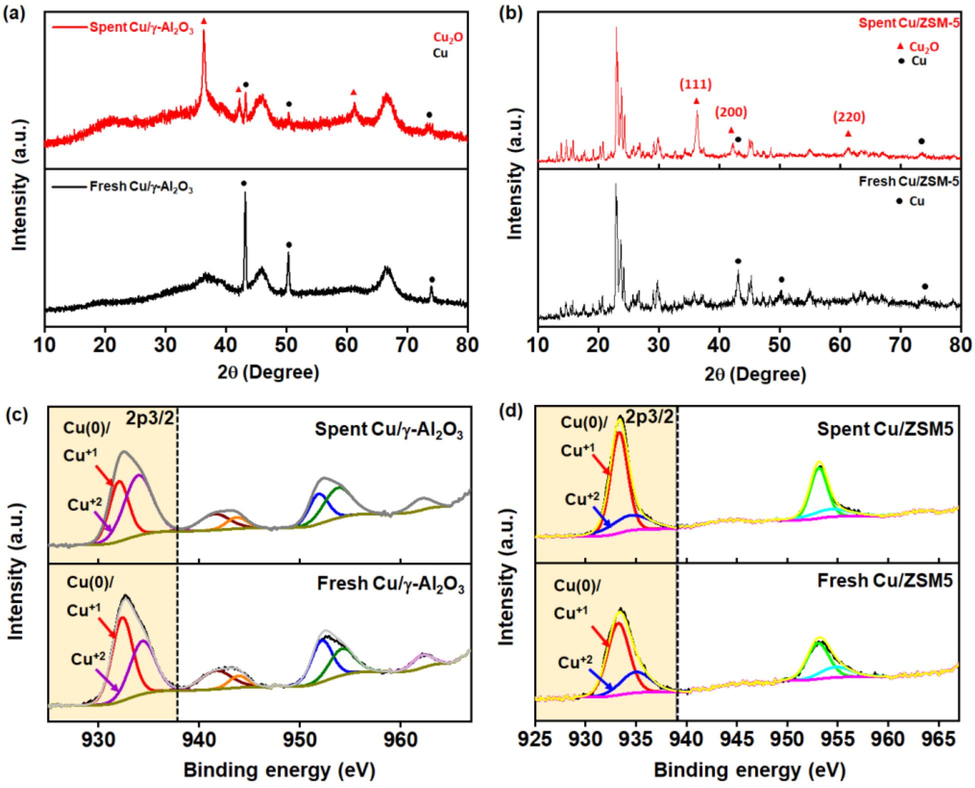

Figure. S13. XRD and XPS profiles of the fresh and spent catalysts, (a) and (c) Cu/γ-Al_2O_3; (b) and (d) Cu/ZSM5. The spent catalysts were collected after the NTP+CO_2+H_2O reaction run over those catalysts at constant SIE of 5.7 J/mL under 1:1 ratio of CO_2 and H_2O feed with 100 mL/min total flow rate for 600 minutes.

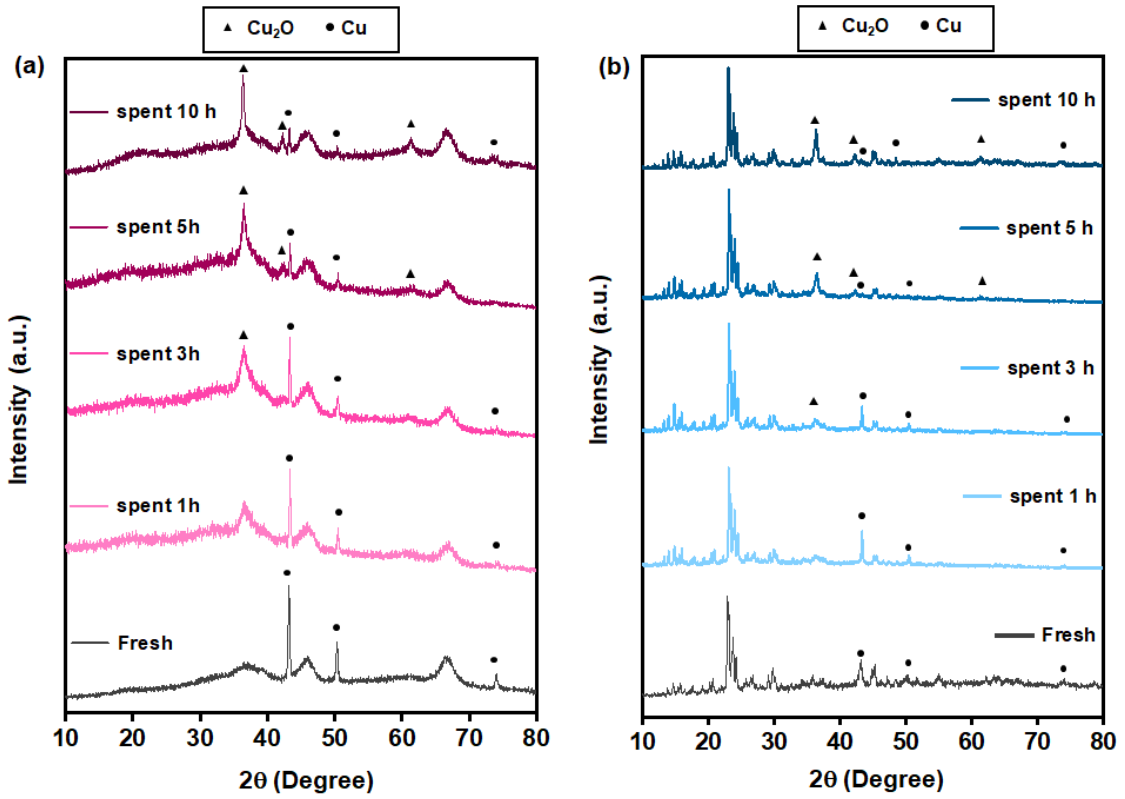

Figure. S14. XRD profiles of (a) Cu/γ-Al_2O_3 and (b)Cu/ZSM5 catalysts spent for 1 h, 3h, 5h and 10h respectively on a contineous NTP+CO_2+H_2O feed under constant SIE of 5.7 J/ml (Experimental condition is same as above).

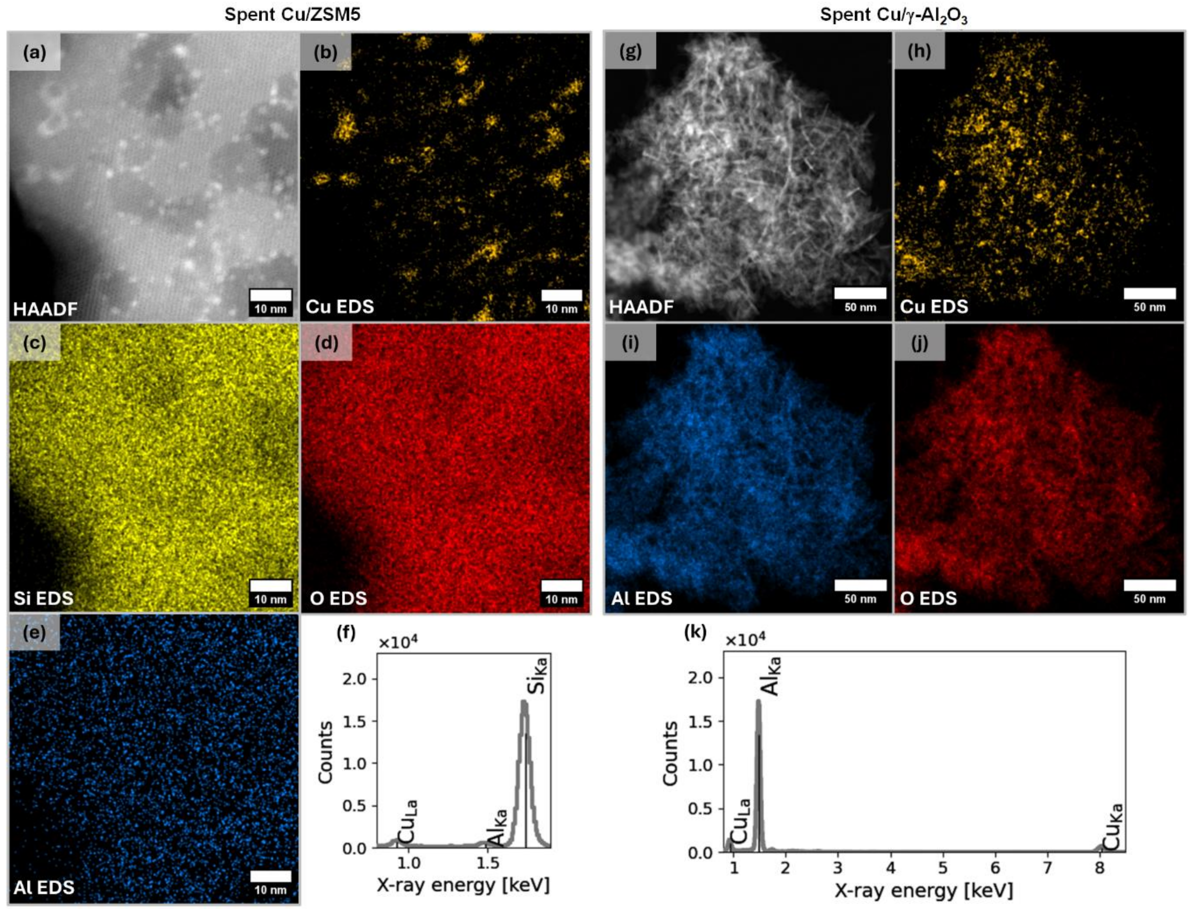

Figure S15. Electron microscopy characterization of spent catalysts. (a - f) Cu/γ-Al_2O_3; (g - k) Cu/ZSM5. Where (a) and (g) are the HAADF STEM images; (b) and (e) are the Cu EDS maps; (c – e) and (i, j) are the EDS maps of the key support elements in spent Cu/ZSM5 and spent Cu/γ-Al_2O_3 catalysts; (f) and (k) are the corresponding EDS spectra. Pores of darker contrast in the ZSM5 support (a) are the result of damage to the support by the electron beam.

10. NTP-Catalytic stability

The catalyst was initially tested in the CO_2+H_2O experiment (as described in Section 3.2 and Figure 4 of the manuscript) on Day 1 for 3 hours, then left overnight under an Ar flow of 50 mL/min. On the following day, the spent catalyst was pre-treated with a 50 vol% H_2/Ar flow (100 mL/min) at a constant specific input energy (SIE) of 14 J/mL for 30 minutes. The pretreated sample was subsequently tested again in the CO_2+H_2O experiment under the same

conditions as outlined in Section 3.2 of the manuscript. This procedure was repeated for three consecutive days, and the results are presented in the table below.

Table S4: Results of NTP-catalytic CO_2+H_2O experiment.

**Catalyst**	**Set**	**SIE (J/mL)**	**CO_2 conv (%)**	**CO yield (%)**	**CH_4 yield (%)**	**H_2 concentration (ppm)**
Cu/γ-Al_2O_3	Day-1 (fresh sample)	5.7	18	16.7	0.14	2400
	Spent-Day-2	5.7	18.3	16.9	0.10	2329
	Spent-Day-3	5.7	17.8	16.2	0.15	2289
Cu/ZSM-5	Day-1 (fresh sample)	5.7	17	16.3	0.10	2500
	Spent-Day-2	5.7	16.6	15.7	0.11	2413
	Spent-Day-3	5.7	16.7	15.6	0.08	2394

Reaction condition: Total flow rate: 100 mL/min, CO_2:H_2O = 1:1, each 2 vol%, Frequency = 27 kHz.

14. ***In-situ*** **DRIFT-MS data for all 4 catalytic system under NTP ON and H_2O IN/OUT condition.**

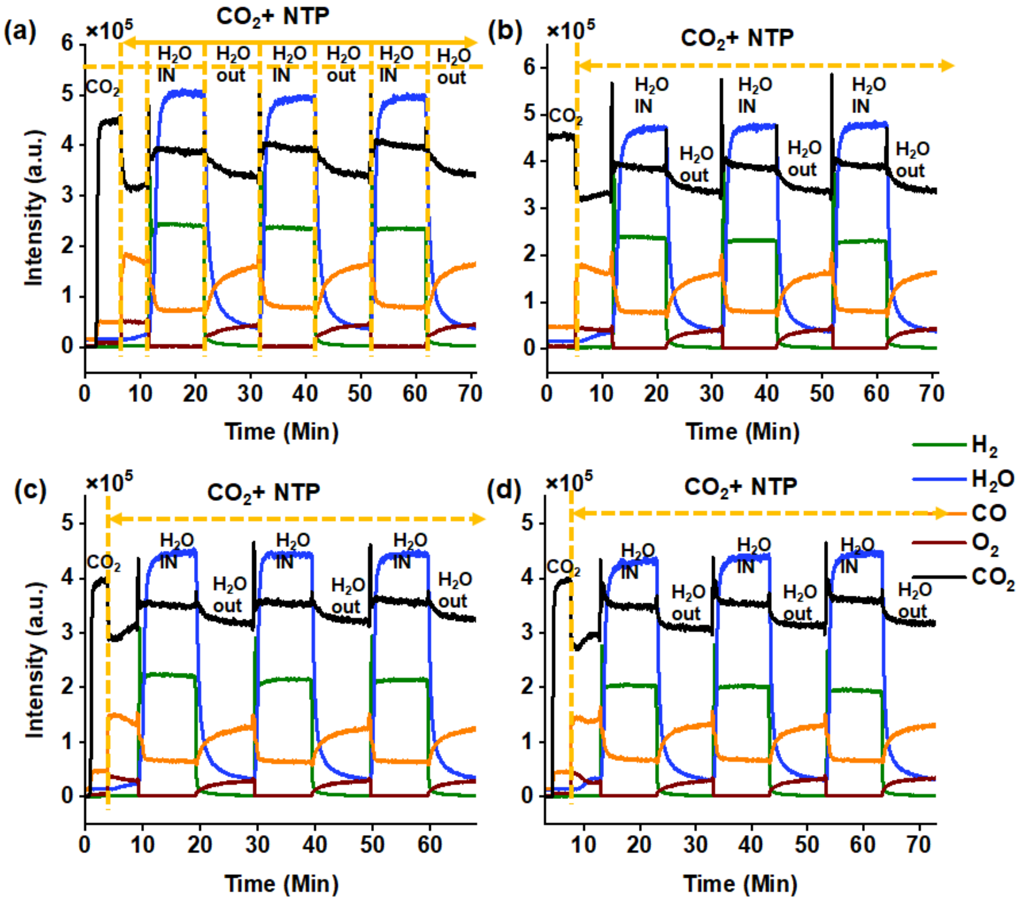

Figure S16. MS signals collected from in-situ DRIFT cell as a function of time during H_2O IN and OUT experiment on different catalyst surface, (a) Cu/ZSM5 (b) Cu/γ-Al_2O_3 (c) Cu/CeO_2 and (d) Cu/TiO_2, under NTP ON condition. Experiment condition: CO_2:H_2O = 1:1, Total flow rate = 50 mL/min, V_{P-P} = 10 kV, Frequency = 27 kHz.

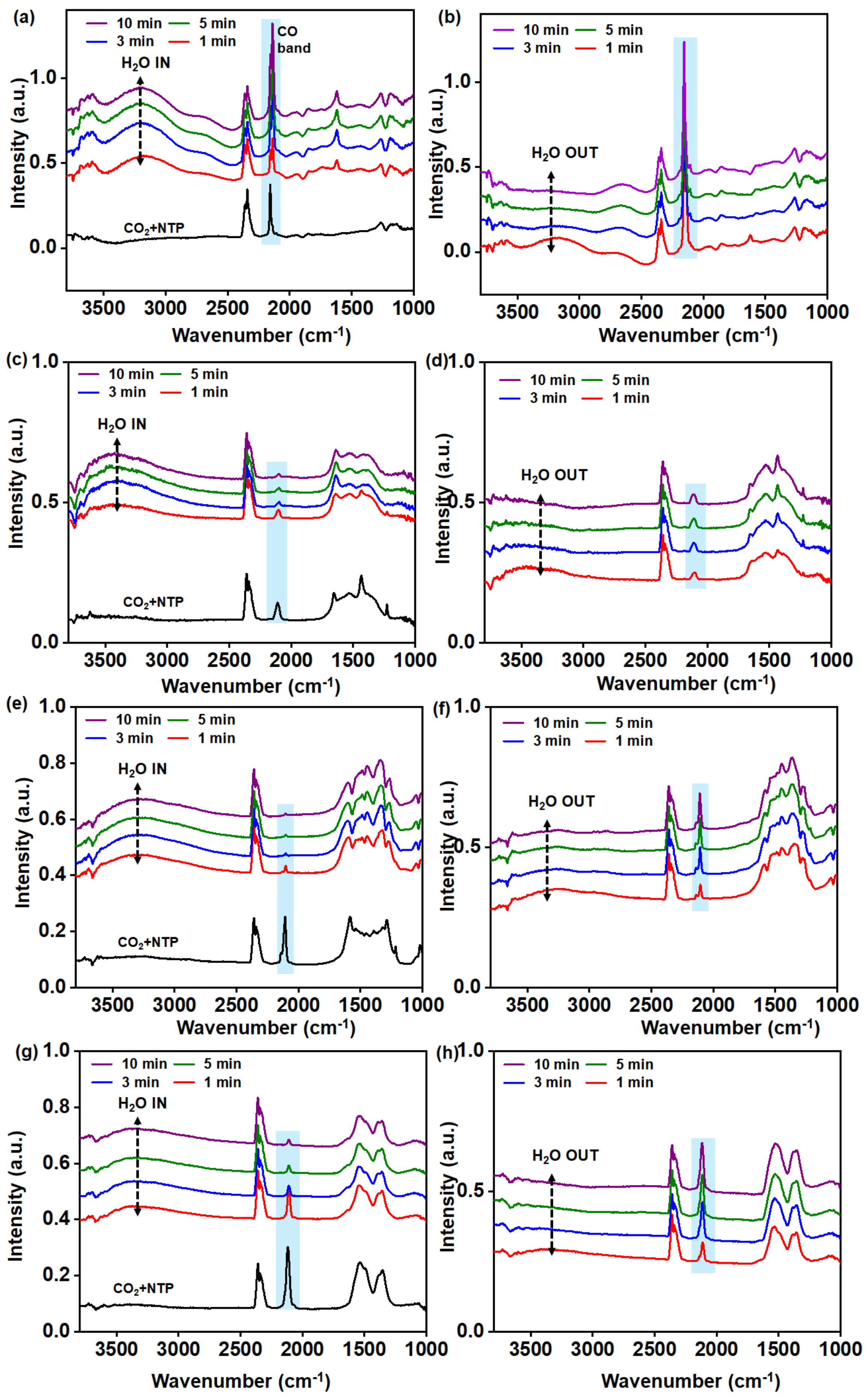
(a)
10 min
5 min
3 min
1 min
CO band
H2O IN
CO2+NTP
Intensity (a.u.)
Wavenumber (cm-1)
(b)
H2O OUT
(c)
(d)
(e)
(f)
(g)
(h)

Figure. S17. In-situ DRIFT spectra (scanned at different time intervals) of adsorbed species on supported Cu catalysts (a),(b) Cu/ZSM5; (c), (d) Cu/γ-Al_2O_3; (e), (f) Cu/CeO_2 and (g),(h) Cu/TiO_2, under 2 different conditions: H_2O IN to the CO_2/Ar feed under NTP ON condition (CO_2:H_2O = 1:1, 2 vol% each +Ar) and H_2O OUT from CO_2/Ar feed under NTP ON condition (2 vol% CO_2/Ar), respectively (Experiment condition: Total flow rate = 50 mL/min, NTP ON: V_{P-P}= 10 kV, Frequency = 27.5 kHz).

11. CO-TPD data analysis for all 4 catalysts

The TPD profiles show 2-3 CO desorption peaks in the range of <250 °C, 250-400 °C and 450-700 °C depending on the catalyst used. The lower temperature peak (<250 °C) indicates the weak interactions between CO and the Cu active sites followed by the moderate and strong interactions at 250-400 °C and 450-700 °C region. Cu/ZSM5 is found to have the highest CO coverage with total amount of 0.319 mmol/g of CO adsorbed followed by Cu/γ-Al_2O_3 (0.276 mmol/g), Cu/TiO_2 (0.135 mmol/g) and Cu/CeO_2 (0.107 mmol/g) respectively.

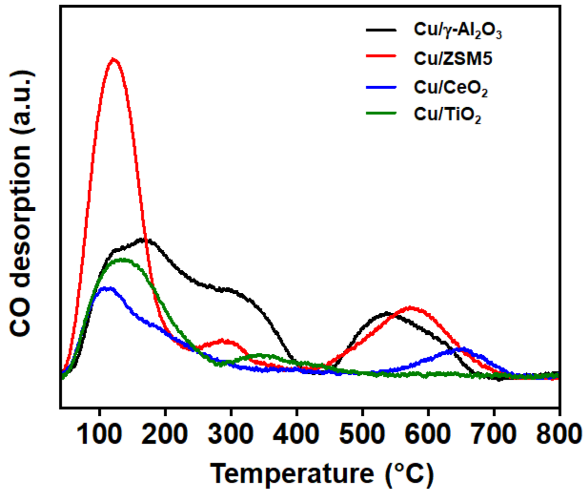

Figure S18. CO TPD profiles of all the fresh catalysts.

12. A deep insight into the *In-situ* DTIFT-MS characterization data for Cu/ZSM5 and Cu/γ-Al_2O_3

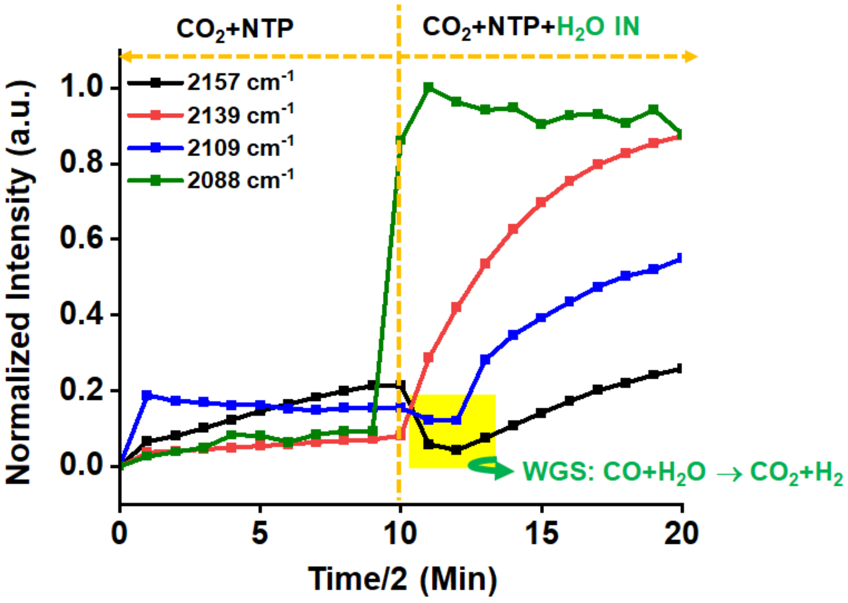

Figure S19. The integrated DRIFT peak areas of different CO species adsorbed on Cu site at Cu/ZSM5 catalyst and how they change on a transition from CO_2+NTP to CO_2+NTP+H_2O IN condition during the 1st cycle of *in-situ* DRIFT experiment (experimental condition stated as above).

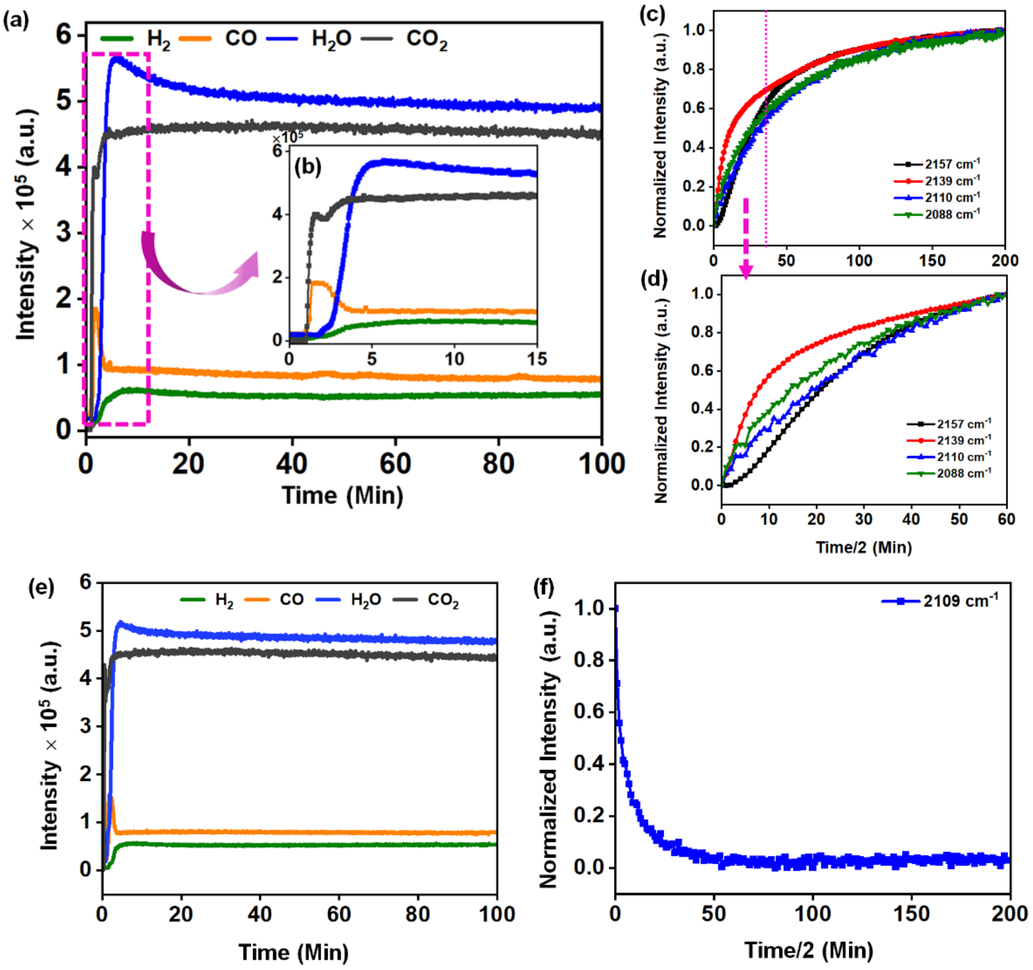

Figure. S20. (a) The MS signals of reactant and product species with respect to the time of stream of CO_2 and H_2O under constant NTP ON condition on Cu/ZSM5 (CO_2:H_2O = 1:1, 2 vol% each +Ar, Total flow rate = 50 mL/min; NTP ON: $V_{P\text{-}P}$= 10 kV, Frequency = 27.5 kHz) (b) Zoomed in figure of (a) within 15 minutes of run (c) Corresponding integrated DRIFT-CO peak areas adsorbed at different wavenumbers at Cu/ZSM catalyst (d) Zoomed in area of the plot (c) up to 30 minutes of run (e) The MS signals of reactant and product species with respect to the time of stream of CO_2 and H_2O under constant NTP ON condition on Cu/γ-Al_2O_3 (CO_2:H_2O = 1:1, 2 vol% each +Ar, Total flow rate = 50 mL/min; NTP ON: $V_{P\text{-}P}$= 10 kV, Frequency = 27.5 kHz) (f) Corresponding integrated DRIFT-CO peak areas adsorbed at 2109 cm^{-1} at Cu/γ-Al_2O_3 catalyst surface.
